# A Review of Disintegration Mechanisms and Measurement Techniques

**DOI:** 10.1007/s11095-017-2129-z

**Published:** 2017-03-01

**Authors:** Daniel Markl, J. Axel Zeitler

**Affiliations:** Department of Chemical Engineering and Biotechnology, University of Cambridge, Philippa Fawcett Drive, Cambridge, CB3 0AS UK

**Keywords:** disintegration, dissolution, *in-situ* monitoring, liquid penetration, modelling, solid dosage forms, swelling

## Abstract

Pharmaceutical solid dosage forms (tablets or capsules) are the predominant form to administer active pharmaceutical ingredients (APIs) to the patient. Tablets are typically powder compacts consisting of several different excipients in addition to the API. Excipients are added to a formulation in order to achieve the desired fill weight of a dosage form, to improve the processability or to affect the drug release behaviour in the body. These complex porous systems undergo different mechanisms when they come in contact with physiological fluids. The performance of a drug is primarily influenced by the disintegration and dissolution behaviour of the powder compact. The disintegration process is specifically critical for immediate-release dosage forms. Its mechanisms and the factors impacting disintegration are discussed and methods used to study the disintegration *in-situ* are presented. This review further summarises mathematical models used to simulate disintegration phenomena and to predict drug release kinetics.

## INTRODUCTION

Solid dosage forms, such as tablets and capsules, still represent the most widespread technology to orally administer active pharmaceutical ingredients (API) to the patient. Within this group disintegrating tablets constitute by far the bulk of pharmaceutical products. By choosing suitable chemical and physical properties tablets can be formulated to either release their API immediately following oral administration (immediate-release tablets) or to modify the drug release profile with the aim to achieve improved therapeutic efficacy, reduced toxicity, and improved patient compliance and convenience (modified release tablets) [1]. Immediate-release tablets are designed to fully disintegrate and dissolve upon exposure to physiological fluids within a short period of time (2.5 to 10 min) [2]. Such a fast disintegration is even more important for orally dispersible tablets, which are designed to disintegrate in the mouth in less than a minutes before swallowing [3]. Such formulations are particularly important where a rapid onset of action is desired, *e.g.* for analgesics [4] or to enable enhanced bioavailability of a poorly soluble drug substance [5]. In contrast, in modified - release tablets the API release may be designed to be gradual in order to achieve slow and sustained dissolution in, or selective absorption across, the gastrointestinal (GI) tract, and/or resulting in a delayed onset time. Such modification of the drug release can be achieved either by embedding the API in a polymer matrix that dissolves or swells at a slower rate than the drug or by means of a suitable polymer coating that acts as a mass transfer limiting barrier [1]. It is common practice to estimate the *in-vivo* performance of a drug product on its *in-vitro* drug release profile by establishing empirical *in-vivo* *in-vitro* correlations during the pharmaceutical product development. However, such empirical dissolution models have a number of inherent drawbacks [6, 7], including that i) the elucidation of the underlying mass transport mechanisms is not possible; ii) not a single characteristic parameter of the dosage form is related to the intrinsic dissolution rate of the drug; and iii) the generality of such empirical models is limited. Therefore, these studies do result in incomplete process and product understanding.

In the majority of cases, the therapeutic dose of a drug is relatively small and therefore the API has to be mixed with suitable excipients to achieve a desired fill volume that allows for compression of the powder mixture into a suitably sized tablet. Properties of the API, such as small particle size [8, 9] and needle-like morphology [10, 11] can lead to processing limitations such as poor flowability [12], difficulties with blending [9] as well as undesirable adhesion [13] to surfaces such as tablet punches or feeder walls [14]. These issues are addressed by selecting an appropriate processing route and/or by adding agents like glidants, lubricants or surfactants [15–18]. The admixture of such excipients is essential to process most APIs and to assure a high product quality [19]. However, embedding the drug in a complex matrix typically reduces its bioavailability, and, in the case of immediate-release tablets, it commonly delays the onset of dissolution. Disintegration agents are therefore added to the formulation, which promote the break up of the tablets into small granules and their constituent particles and thus enable a faster liberation of the drug particles from the tablet matrix leading to an increase in surface area for subsequent dissolution. The most widely used disintegrants are synthetic polymers such as crospovidone (XPVP), croscarmellose sodium (CCS) and sodium starch glycolate (SSG) [5, 20–22]. Given that in immediate-release tablets disintegration is a necessary requirement for dissolution, the disintegration performance has a direct impact on the therapeutic effect of the medication and must be assessed, and ideally quantified, using specifically designed disintegration tests.

**Fig. 1:**
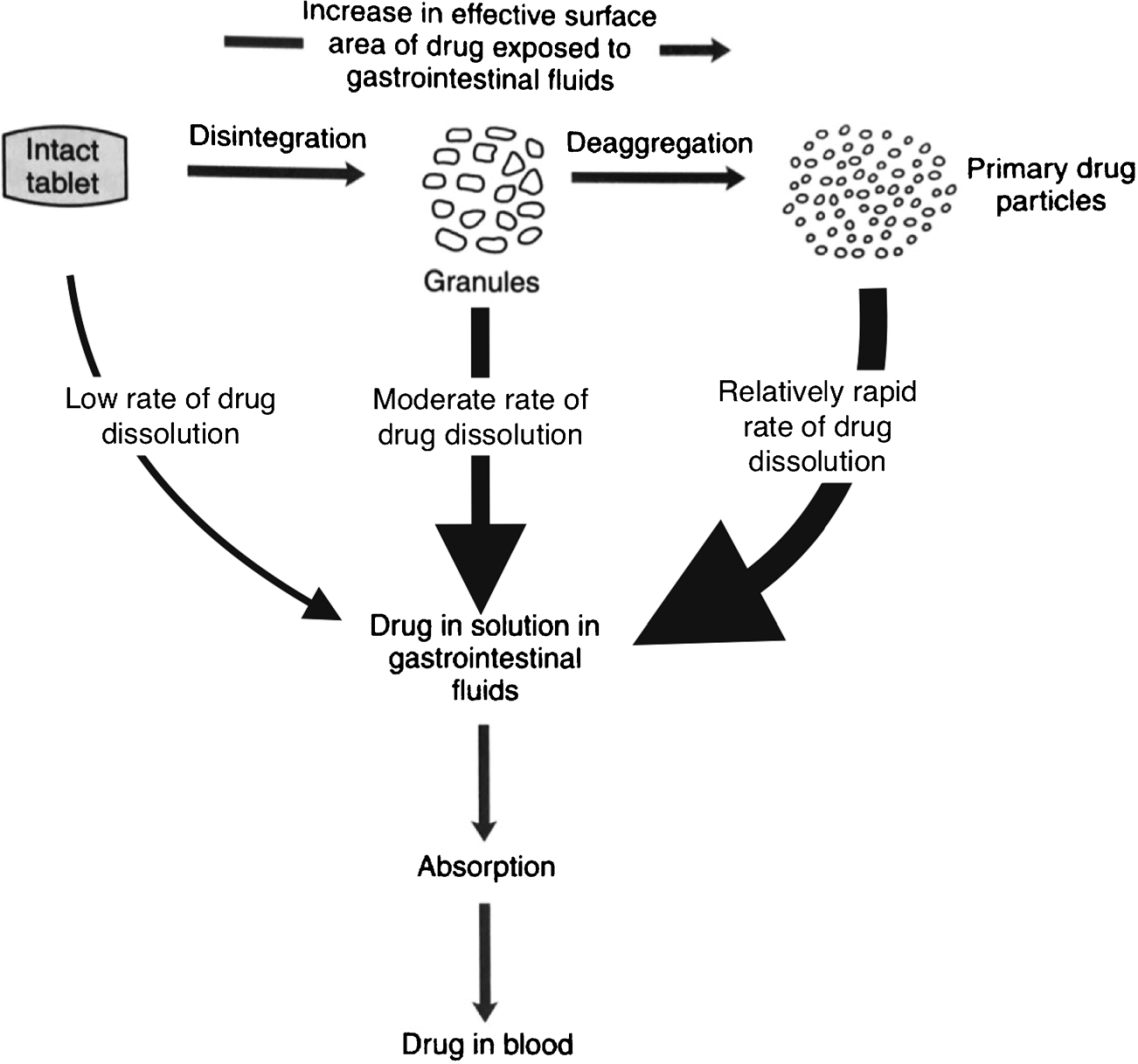
Schematic of the drug release process from a tablet (modified from [207]).

The disintegration process is an integral step in ensuring, and indeed maximising, the bioavailability of the API from the majority of solid dosage forms. With the exception of diffusion - controlled matrix systems, in tablets the wetting and subsequent disintegration of the powder compact is the first step towards the liberation of the API from the dosage form. Without disintegration only the API near the surface of the tablet would be able to dissolve and hence the reproducible and full disintegration of the tablet upon exposure to the dissolution medium is of critical importance to achieve a reliable clinical performance of the dosage form (Fig. 1).

Given the central role of the disintegration process for the therapeutic success of the dosage form it is somewhat surprising that the mechanistic understanding of this process has not received more attention over the past 50 years. In our view this lack of understanding can be explained by a combination of the complexity of the disintegration process paired with the absence of quantitative measurement techniques to accurately describe the disintegration process in sufficient detail. Compared to other scientific disciplines that deal with similar processes the mechanistic understanding of pharmaceutical disintegration poses a range of significant problems: i) There is an enormous variety of disintegrating matrices of interest. Essentially each tablet formulation is unique from a chemical point of view given the vast range of API properties and the wide range of excipients that are in common use. ii) Some formulations contain excipients that swell significantly over time with exposure to the dissolution medium, resulting in strongly non-linear time and temperature dependence of the swelling process. iii) The process route (direct compaction, dry or wet granulation, compaction conditions) has a significant impact on the tablet microstructure and changes in these parameters are common during the pharmaceutical development process. In many cases the final microstructure of the dosage form is only defined by the production scale process development just before the product is produced commercially and where no significant changes in formulation are possible any longer given the regulatory filing requirements and the pressure to minimise the time to market. iv) Changes in the physical properties of the supplied excipient have traditionally not been as tightly controlled compared to the chemical quality and impurity profiles. In addition, different batches of API can exhibit changes in particle size and morphology. v) Prior to the quality by design (QbD) initiatives changes to the process parameters during commercial production of a marketed product were extremely costly and hence a better understanding of the microstructure of the dosage form was not of much commercial advantage to the industry as batches that failed disintegration were likely to be discarded.

Against the background of such formidable challenges it is easy to understand that for a long time there was little motivation to understand the complex physics of tablet disintegration from a commercial perspective. It is well understood that the drug release kinetics is a, if not the, critical link between the solid dosage form and the API plasma concentration. Given there are numerous sophisticated highly accurate methods available to quantify the amount of API released form a dosage form over time during *in-vitro* dissolution tests it makes perfect sense that the detailed understanding of the dissolution process and the field of *in-vitro* *in - vivo* correlations has attracted such strong interest. The need to develop a sound understanding of dissolution also explains why there has been relatively little activity in advancing the detailed insight into the disintegration process. However, in this context it is also crucial to highlight the lack of suitable analytical technologies to reliably identify, measure and quantify the complex mass transport processes and mechanical changes in a tablet sample during disintegration. In the absence of such measurement technologies it is clearly not possible to develop accurate mechanistic models – and it is only through the understanding of the disintegration process that it is possible to fully quantitatively describe the dissolution of API as it is necessarily the first step of drug release from a disintegrating matrix (Fig. 1). Whilst the assumption of rapid and full disintegration might be justified in the majority of cases there is sufficient anecdotal evidence that a substantial amount of batch failures in immediate-release dosage forms have their root cause in poor, and unexplained, disintegration behaviour.

The first official disintegration test was published in the Swiss pharmacopeia in 1934 [23]. The method specified that a tablet has to be placed in a 100 mL conical container, then 50 mL of water at 37 ° C was added, and upon exposure of the tablet to water the container was shaken periodically. The disintegration time had to be 15 min or less. Over the years a number of different methods were developed that were based on the same concept but saw some variation in terms of the mechanics of the testing instrument by using tubes [24], a rolling drum [25] or meshes [26]. In addition, there were variations in terms of the disintegration liquid by introducing simulated gastric juice [27] to better mimic the actions in the body on the tablet [28]. Filleborn developed an artificial stomach by simulating the pH, presence of food, peristalsis, volume of gastric juice and hydrostatic pressure, and emphasised that simple *in-vitro* disintegration tests are valuable only if they can simulate the *in-vivo* conditions [29]. However, the disintegration test that is required today by the respective pharmacopoeiae [30–32] does not differ significantly in terms of the measurement concept developed for the very first test that was introduced in 1934: a tablet is placed within an open ended tube on a wire mesh that is fitted at one of its ends. The tube with the tablet is then mounted such that it can be periodically moved up and down in a 1 L beaker of water, simulated gastric juice or simulated intestinal fluid at 37 ± 2 ° C for a predetermined time. After the exposure period the tube is checked for the presence of the sample specimen. If a palpable core is still present the test is considered to have failed. This type of test was reviewed in detail by Donauer and Löbenberg [33]. Whilst the test is overall suited to establish whether or not a tablet fully disintegrates within a given exposure period, or how much time is required to disintegrate a tablet, such traditional disintegration testing does not provide any insight into the mechanism of tablet disintegration. The results of the disintegration test are used nonetheless to assess whether the dosage form meets the requirements of the respective pharmacopoeia even though it yields little fundamental information about the drug release behaviour of the dosage form. As outlined above, a detailed understanding of the underlying disintegration mechanisms which occur when the tablet comes in contact with the physiological fluid is highly desirable. Such understanding requires the development of mechanistic models which describe the fundamental mechanisms based on quantitative disintegration and dissolution data. Significant advances in analytical techniques over the past years enabled the quantitative investigation of changes in the microstructure during the disintegration of a pharmaceutical tablet. Experimental data from such analytical techniques is the basis for a comprehensive understanding of the functionality of the excipients and the API as well as their influence on the disintegration and dissolution process. The aim of this review is to provide an overview of the mechanism of disintegration, to present different methods used for *in-situ* monitoring of the microstructural changes of pharmaceutical powder compacts, and to summarise the existing models used for describing the different disintegration phenomena.

## MECHANISM OF TABLET DISINTEGRATION

**Fig. 2:**
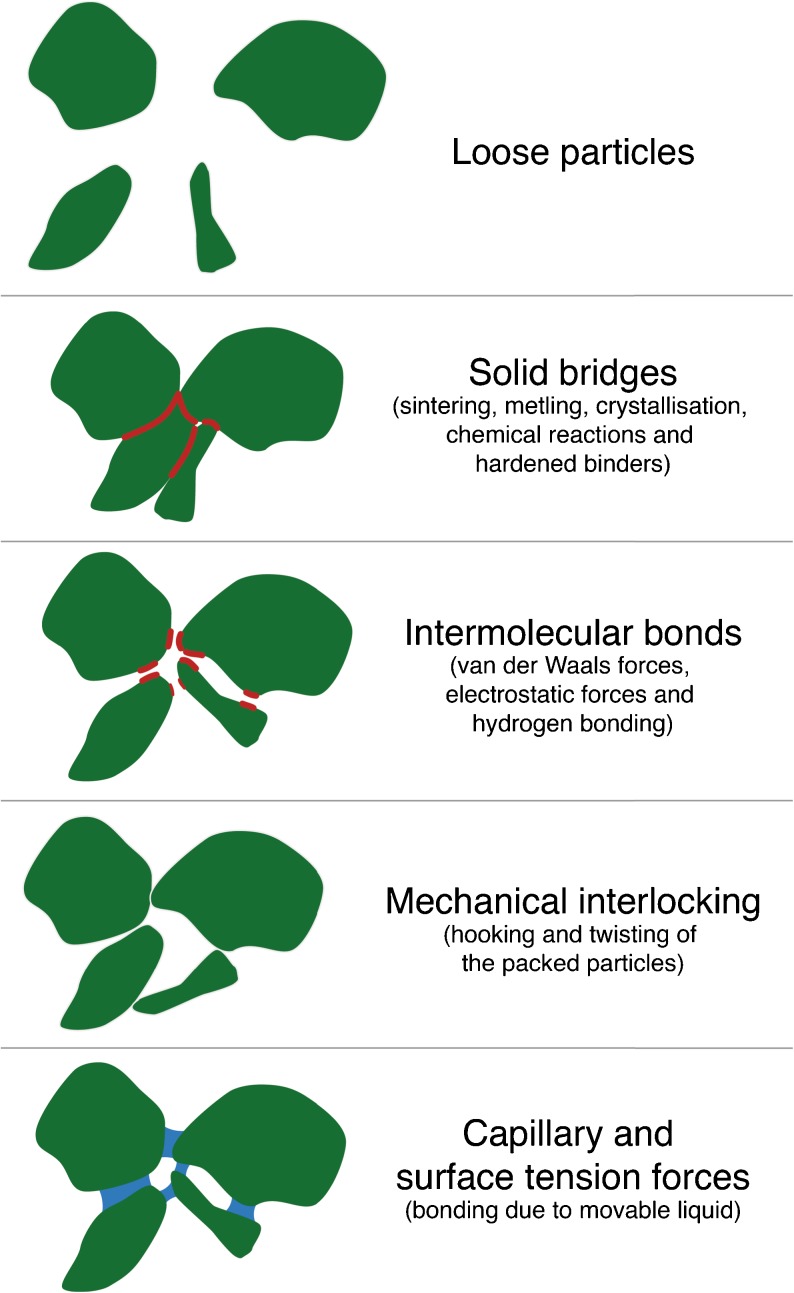
Overview of particle bonds. The bonding surface areas are highlighted in *red*.

Disintegration refers to the mechanical break up of a compressed tablet into small granules upon ingestion and therefore it is characterised by the breakdown of the interparticulate bonds, which were forged during the compaction of the tablet. It is hence a good starting point to briefly reflect on the physical changes that take place during the compaction process: i) particle rearrangement, ii) elastic deformation, iii) plastic deformation, and iv) fragmentation of particles, as well as v) the formation of interparticulate bonds [34]. Steps ii) to v) may have a direct influence on the disintegration of the powder compact. The reduction of the compact volume is performed by the reversible elastic or by the irreversible plastic deformation. After an initial volume reduction the particles can be divided-up into smaller particles, a process that is also called fragmentation. These smaller particles may then undergo further elastic and/or plastic deformation. When the particles come into close proximity to each other they can form interparticulate attraction bonds, such as intermolecular bonds, solid bridges and mechanical interlocking (Fig. 2) [34]. Naturally, the bonding surface area limits the maximum tensile strength that can be achieved for the powder compact. Intermolecular bonds in general, and van der Waals forces in particular, dominate the cohesive characteristics of many direct compression binders, such as microcrystalline cellulose (MCC, Avicel®) and lactose. Solid bridges are defined as the contact at an atomic level between adjacent surfaces of particles and thus, these forces act up to a distance of 1 nm. Mechanical interlocking is the hooking and twisting together of packed particles. A high compaction load is required to generate mechanical interlocking and this bonding mechanism depends on the shape and surface structure of the particles, *i.e.* long needles and irregular particles have a higher tendency to hook and twist together during compaction compared to smooth spherical particles [34]. Nyström *et al.* [34] and Adolfsson *et al.* [35] showed on the basis of the tensile strength of tablets that the bonding structure and the bonding mechanisms depend on the chemical structure, volume reduction behaviour (*i.e.* elastic deformation, plastic deformation and particle fragmentation) and particle size. Furthermore, during disintegration the liquid penetrating the powder builds capillary bridges, which cause an attractive force of adjacent particles. A force has to be generated during disintegration which surpasses the interparticulate forces and disrupts the bonds. The actual bonding mechanisms and bonding surface area thus have a direct impact on the disintegration process.

**Fig. 3:**
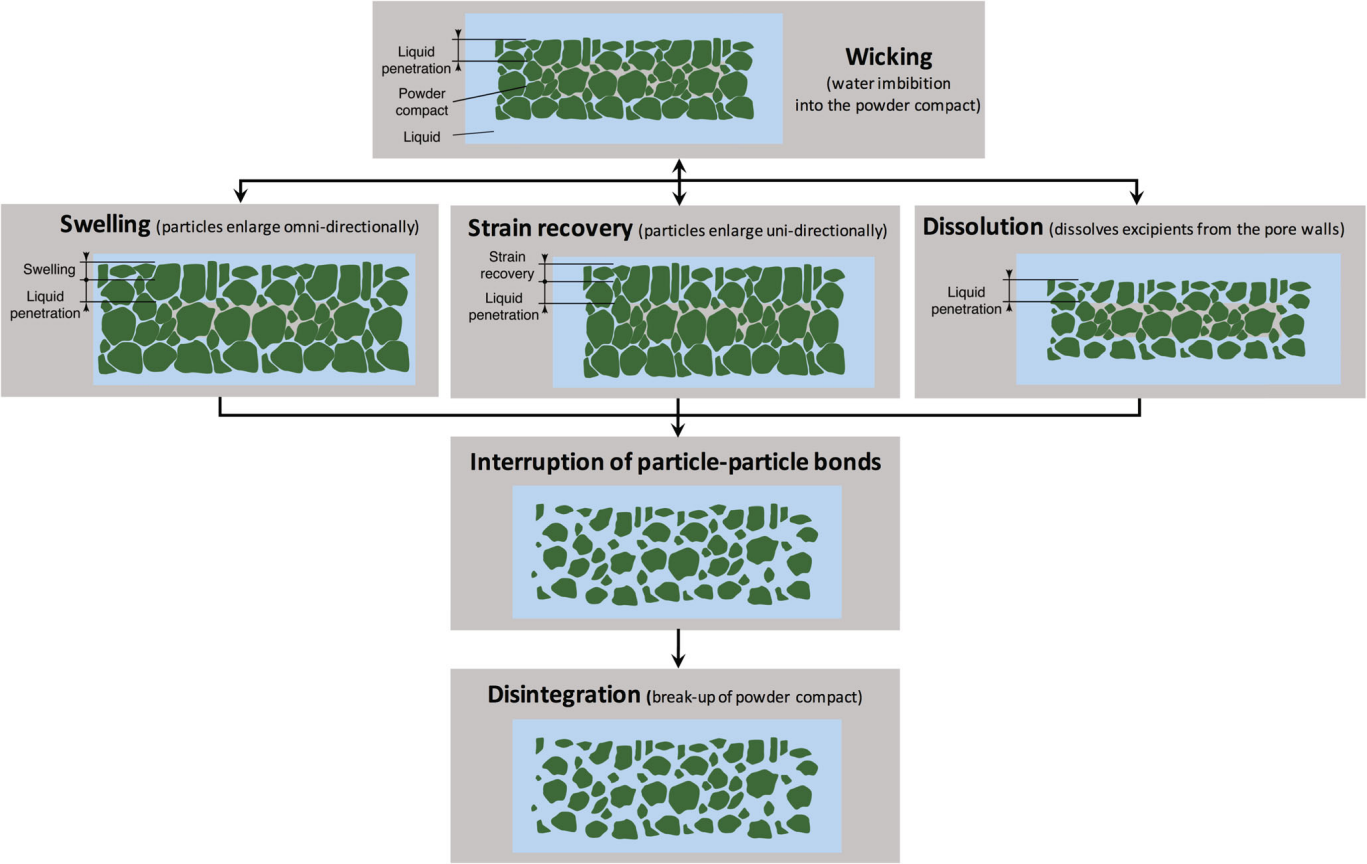
Overview of mechanisms involved in disintegration of pharmaceutical powder compacts.

The first and often the rate-determining step in disintegration is the liquid penetration in the porous powder compact [36]. Liquid penetration does not directly build up the pressure which is necessary to rupture the particle-particle bonds, but it is a prerequisite to initiate other mechanisms like swelling (see Fig. 3).

Swelling is one of the most accepted mechanisms involved in disintegration [37–39]. The swelling is the omni-directional enlargement of particles, which build up pressure, push apart adjoining particles, leads to exertion of stresses on the overall systems and finally breaks up the tablet [21]. The dissolution fluid in itself exerts a force in the tablet pores, but this force alone can be too low to be effective, particularly if the bonds between the solid particles are strong. In the presence of a disintegrant, however, the forces exerted by the fluid become appreciable enough to destroy the compact [40].

Another well-known disintegration mechanism is strain recovery. The strain within the tablet is the consequence of forcing macromolecules into a metastable configuration either due to interlocking of the polymer chains or as a result of spontaneous crystallisation during the compaction of a tablet. The stored energy can be released as heat immediately following the compaction or, if this is not or only partially the case, when the polymer comes in contact with a fluid, *i.e.* disintegration medium or physiological fluids. Hydration of the polymer gives rise to sufficient mobility for entropy recovery to take place, and, with that, recovery of the original shape of the polymer molecules [22, 41]. Therefore, strain recovery can be regarded as the reversible viscoelastic process of deformation [42]. It is uni-directional and in the opposite direction of the applied compression force (see Fig. 3). Recently, Desai *et al.* [43] and Quodbach *et al.* [44] investigated strain recovery in more detail and they concluded that one of the disintegration mechanisms of tablets containing XPVP is due to strain recovery.

Moreover, hydration, swelling and strain recovery of many hydrophilic polymers in water changes the mechanical properties of these materials from dry solids to soft and rubbery states. The sorption of water results in a lowered glass transition temperature (*T*_*g*_) of the polymer, which may impact the swelling or strain recovery kinetics. Schott [45, 46] revealed that the kinetics change from first-order to second-order swelling kinetics as the *T*_*g*_ is crossed during swelling and hydration and that an equilibrium is reached when the swelling pressure equals the elastic recovery of the swollen network. In the presence of the strong dipole and high mobility of water molecules interchain macromolecular hydrogen bonds can break, which reduces the interchain attraction and further plasticise the amorphous portion of the polymer. This allows additional chain segments to slip past one another and weaker the cohesive energy between the chain segments of the structure to absorb more fluid. In contrast, the more dense crystalline regions of the polymer contribute far less to swelling as they are less accessible by the water molecules and the cohesive forces between chain segments is higher compared to the amorphous domains. High degrees of crystallinity of such swelling polymers can thus slow down or even prevent disintegration [46].

Independent of whether the volume enlargement of the polymer powder particles is caused by strain recovery, swelling or a combination thereof the strain that develops within the porous tablet matrix is released through the growth of defects into micro-cracks, which in turn increases the (easily accessible) pore space in which water can enter. This process accelerates tablet hydration and, in turn, disintegration.

In addition, the fluid can dissolve or dislodge excipient particles from pore walls, which can significantly affect the porosity and as a result the disintegration performance [22, 47, 48]. Not surprisingly this effect is especially significant for powder compacts incorporating soluble components [22, 49]. As a result the viscosity of the liquid phase and the structure of the porous system can change drastically with time; both effects would impact liquid penetration [50]. Shah and Augsburger [51] investigated the effect of physical differences on the disintegration and dissolution for a disintegrant (XPVP) from different sources embedded in either a soluble or insoluble matrix. They concluded that there is a direct effect of the physical properties of XPVP (including particle size and distribution, surface area, porosity and surface morphology) on the disintegration time and dissolution rate when used in a formulation that was based on an insoluble filler. Even though overall a faster disintegration could be achieved for a formulation using a soluble filler compared to a tablet with an insoluble filler, differences in physical properties of XPVP did not affect the disintegration time. The effect of the solubility of the filler is intuitive in that the filler is typically present at relatively large concentration and so long the dissolution rate of the filler is reasonably high the liquid can easily penetrate into the soluble matrix and hence disintegrate the tablet.

It has further been proposed that exothermic (heat generation) and endothermic (heat absorption) processes can cause, or at least facilitate, the break up of the powder compacts [47, 52]. Specifically, it was hypothesised that the generation of heat may cause localised stress due to the expansion of air retained in the powder compact leading to the break up of the *inter*-particle bonds. It is important to note in this context that the papers by Matsumaru were published in Japanese and hence potentially hard to retrieve from the U.S.A. at the time as evidenced by the fact that Loewenthal cites the Chemical Abstracts service in addition to the original citation in his review. The papers are now readily accessible and closer reading of the work reveals that Matsumaru did not claim that the heat of interaction is a fundamental disintegration mechanism but rather he provided calorimetric data to show that there can be measurable heat upon disintegration [52–58]. The results are in good agreement with the discussion of entropy recovery above. Besides this potential misunderstanding of the literature it is questionable from a physical point of view if the pressure built up in residual air by the change in temperature from such localised stress could ever initiate tablet disintegration. If this would be a significant mechanism, then the heat generated during compression and ejection of the tablet would already disrupt particle-particle bonds, which would lead to the break up of the tablet immediately after compaction [21, 47]. In the light of the limited experimental evidence that has been presented for this hypothesis by just a single research group in the late 1950s and the relatively modest amount of stored energy, that furthermore would need to be released instantaneously to result in any appreciable pressure build up, this mechanism of disintegration should no longer be considered.

More information about the actions of various disintegrants and their mechanisms can be found in several other recent review articles [21, 22, 47].

## FACTORS AFFECTING LIQUID PENETRATION

Disintegration is achieved by the penetration of the physiological fluid into the powder compact and the subsequent disruption of the particle-particle bonds which maintain the structural integrity of the dosage form. Therefore, liquid penetration (or wicking) is one of the key steps involved in the disintegration process. The rate of penetration of liquid into a porous matrix is driven by the interplay between the capillary forces that promote fluid movement towards the interior and the viscous forces that oppose the liquid movement. Liquid retention and flow in unsaturated porous media, where the pores are filled with both liquid and air, are thus driven by the balance between cohesion among the liquid molecules and adhesion between the liquid molecules and the particle surfaces [59].

Therefore, the wicking process in immediate-release tablets is assumed to be driven by capillary action, as it was already suggested in 1955 by Curlin [60] for aspirin tablets. Capillary action is a well studied phenomenon due to its numerous applications, such as in petroleum engineering, in hydrology (*e.g.*, movement of ground water), in consumer products (*e.g.*, marker pens, candle wicks and sponges) or in plants (*e.g.*, transport of water from the roots to the tips). Mathematical models have been well established for some time to describe the volumetric flux in a porous medium. By combining the Hagen-Poisseuille (Eq. 15) and the Young-Laplace (Eq. 16) equations, two such expressions that arewell known in fluid dynamics, the following expression for the volumetric flux *q* of the fluid as a function of the penetration depth, *L*, can be derived (see Appendix A for the derivation):1


**Fig. 4:**
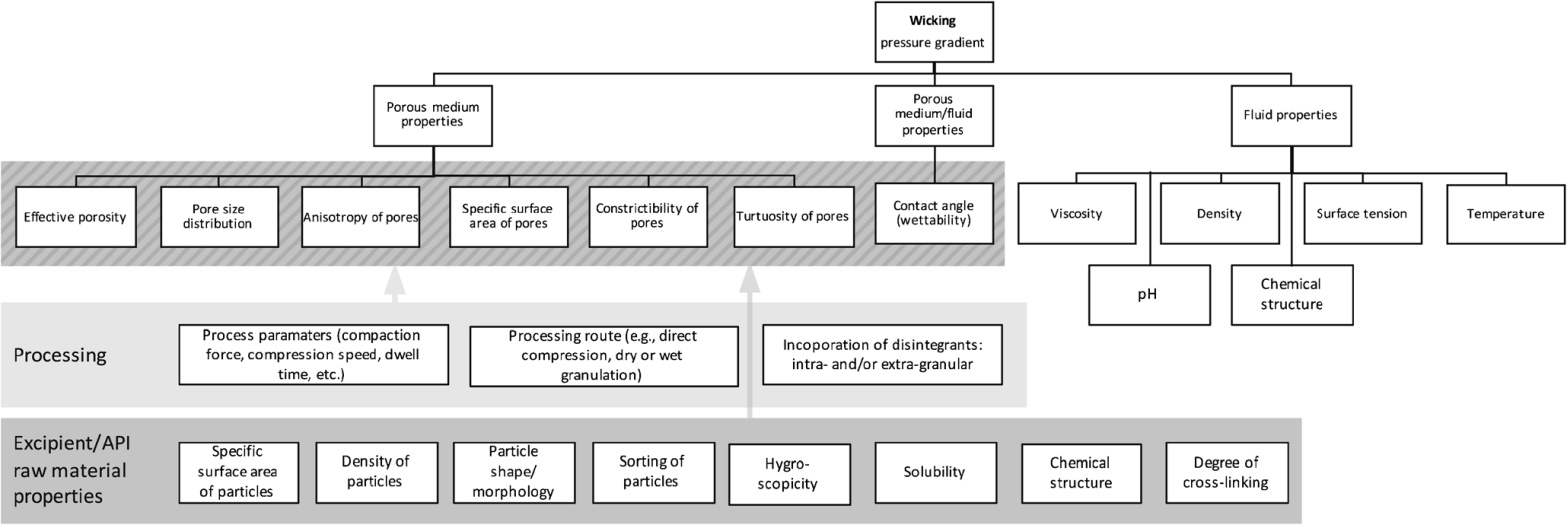
Impact of porous medium properties, fluid properties, processing parameters and routes as well as raw material properties on wicking. The arrows and shaded areas highlight the influence of processing and raw material related properties on wicking.

The dependence of the liquid penetration on the physical properties of the matrix, fluid and fluid/matrix can readily be recognised in the mathematical representation of the volumetric flux (Fig. 4). The relevant fluid properties are surface tension, *γ*, and viscosity, *η*. The contact angle, *θ* (wettability), is a fluid/matrix property and the relevant solid matrix properties are pore size distribution, Δ*β*, and tortuosity, *τ*. *R*_*h*,0_ is the hydrodynamic radius and *R*_*c*,0_ is the capillary radius, which is seen by the liquid meniscus. The capillary force remains reasonably constant, whereas the viscous forces increase with penetration causing a decrease in the overall penetration rate as saturation proceeds. However, the viscous forces along the disrupted pore system may drop due to a disruption of the particles and this in turn can lead to an increase in penetration rate. At the same time, the capillary forces may remain unchanged as the curvature of the meniscus of the advancing liquid front is governed by the dry, undisrupted, pore system [61]. In contrast, the capillary force is influenced by the hydrophilicity (related to the contact angle) of the excipients, discussed by Guyot-Hermann and Ringard [62]. Here the importance of sufficiently well distributed hydrophilic excipients in a tablet was emphasised. Such excipients can convey liquid from the surface to the centre to accelerate disintegration. Although the physical properties of the fluid and the pore structure influences both capillary and viscous forces, once the excipients are selected tablet formulators can only control the pore structure as the physical properties of disintegration liquid are typically not free variables (even though different dissolution media certainly will exhibit a range of viscosities and wetting behaviours).

In pharmaceutical practice the pore structure is often only described by the total porosity, which is the fraction of the volume of voids over the total volume and thus it is a measure for the void space in the material. It was shown that the tablet porosity is one of the most important contributors to the disintegration performance [63] and that it highly depends on the compaction force and compression speed [10, 36, 64–67]. In general, small pores decrease the ability of a fluid to enter the powder compact, whereas a high porosity, associated to a large void space, may lower the force induced by the swelling of excipients. Therefore, a lower swelling force increases the time to break up *inter*-particle bonds [47, 68]. However, the complex pore structure cannot by adequately represented by one single parameter such as the total porosity. Porous media can be more accurately described by a combination of parameters such as characteristic length (effective pore radius in the porous medium), a constriction factor (fluctuation in local hydrodynamic radii), a tortuosity (effective length of the streamlines) and an effective porosity (the ratio of the volume of the conducting pores to the total volume). The characteristic length, tortuosity and constriction factor are direction dependent descriptors of the pore structure, and an anisotropic permeability behaviour of powder compacts is not uncommon [69–72]. In line with such behaviour it was shown in a number of studies that the density of tablet matrices is often unevenly distributed (*i.e.* density increases with radius for standard biconvex tablets) [73–75].

**Fig. 5:**
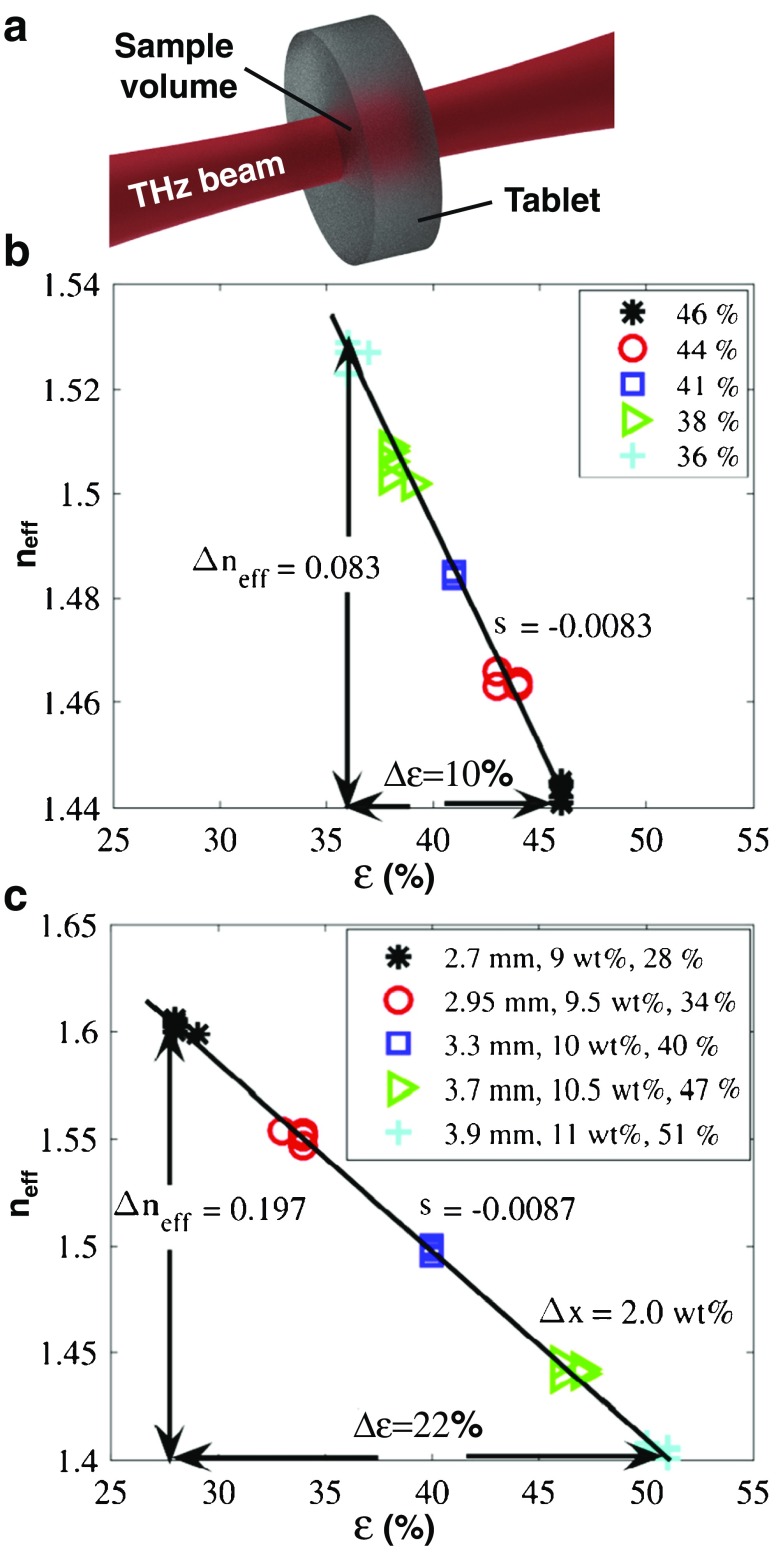
Method to determine the porosity of a flat faced tablet by THz-TDS. Tablets of MCC and indomethacin were varied either in porosity *ε*, height, or API mass fraction *x* as specified in the legend. (**a**) Schematic of the terahertz transmission measurement to measure the effective refractive index, *n﻿*_eff_, of a tablet. (**b**,**c**) Effective refractive index measured by THz-TDS as a function of the porosity of a tablet. The porosity was calculated from the relative and apparent densities of the sample. The API mass fraction in (**a**) was kept constant at 10 wt% (modified from [84]).

These key characteristics that describe the pore network structure can often be extracted sufficiently well from high-resolution three-dimensional X-ray computed microtomography (XμCT) images of porous media [76, 77], although the detectable pore sizes are often limited by the pixel size of the image and samples have to be taken from the dosage form to achieve the highest resolution (> 0.5 μm for the most commonly used commercial bench-top instruments) [78]. In addition, traditional porosimetry techniques can be employed to measure the porosity directly using liquid or gas intrusion [79, 80]. An alternative approach to rapidly and non-invasively measure the bulk porosity of whole tablets was recently demonstrated by Bawuah *et al.* [81] using terahertz time-domain spectroscopy (THz-TDS) [82, 83]. The analysis of porosity from terahertz measurements is based on its relation to the effective refractive index of the probed tablet (Fig. 5). The method was demonstrated by measuring the porosity of thin flat faced tablets [81] containing pure MCC as well as flat faced tablets made of MCC and an API [84]. This was further developed to analyse thicker biconvex tablets [85], which led to the analyses of immediate-release tables consisting of a complex formulation [86]. This study is discussed at the end of this section (Fig. 5).

Besides the pore structure itself further factors need to be taken into account when considering the liquid penetration into a porous medium. The capability of a porous medium to transmit fluid is typically summarised by its permeability, *K* (as defined in Darcy’s law [87]), which is therefore a fundamental attribute of the performance of the disintegrating tablets. The permeability is however closely tied to the pore structure of the powder compact (the derivation is given in Appendix B):

2 $$ K=\frac{1}{8\tau}{\displaystyle \sum_{j=1}^M}\Delta {\beta}_j{R}_{h, j}^2. $$

**Fig. 6:**
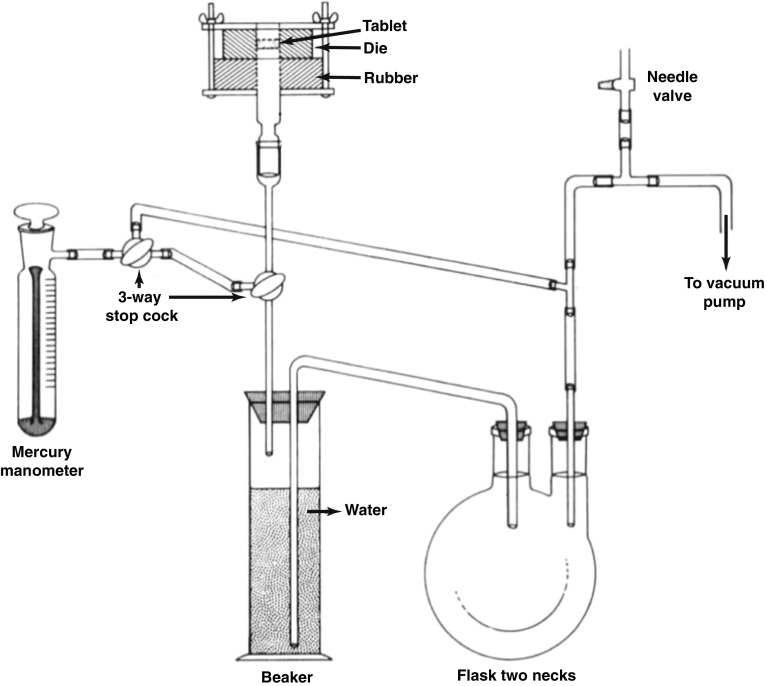
Air permeability apparatus from Lowenthal and Burrus [88]. The impinger beaker was filled with freshly boiled, cooled water. The tablet was sealed to the rubber stopper and the 3-way stop cock were opened prior to the measurement.

One of the first experimental approaches to measure air permeability of a tablet (Fig. 6) was presented by Lowenthal and Burrus [88]. The system consisted of a vacuum rig with the tablet sealed into a rubber stopper that separated the vacuum from the atmosphere. The rate of air permeating through the tablet was measured by the amount of water that was displaced in the connected impinger over time whilst also recording the pressure drop. The authors then calculated the mean pore diameter from the air permeability measurement using the Kozeny-Carman equation. A similar procedure was presented by Alderborn, Duberg and Nyström [89] to determine the specific surface area of pharmaceutical tablets from air permeability measurements. However, these measurements provide an accurate measurement for the permeability with air and it is not trivial to extend the method to measure liquid penetration into the powder compact due to the complex interplay between liquid penetration kinetics, swelling and dissolution, which result in a time- and spatially-dependent permeability.

The interdependence between permeability of a tablet and its pore structure, and thus porosity, was studied by Ganderton and Fraser [61] for different formulations ranging from aspirin, lactose, magnesium carbonate, calcium phosphate to phenindione and sucrose tablets. They emphasised the impact of tablet compaction pressure, particle size and granulation on the porosity and permeability. In particular, they reported that almost impermeable structures were formed from fine particles of aspirin and phenindione which resulted in the lowest water penetration rate. In contrast, formulations containing lactose resulted in the most permeable tablets and yielded the fastest liquid penetration.

As already mentioned above, the fluid composition can significantly influence the disintegration of powder compacts [90]. Biorelevant media considerably differ in viscosity, contact angle and surface tension and hence the liquid penetration kinetics is affected by the choice of medium [91]. The majority of studies focused on using water as the disintegration medium and thus may lack physiological relevance as most of the tablets are designed to disintegrate in the gastric juice and not in water. This was already highlighted by Abbott *et al.* in 1959 [92], where the authors compared the disintegration of commercial tablets in simulated gastric juice with the same experiment carried out with pooled human gastric juice. The *in-vitro* disintegration was prolonged in human gastric juice and Abbott *et al.* assumed that this was due to a change of the liquid properties: a higher viscosity of the medium leads to a slower disintegration and a lower surface tension of the medium results in more rapid disintegration [36, 92, 93]. A more viscous fluid may promote adhesion between larger particles, and thus counteract the swelling mechanism of disintegrants. Moreover,depending on the temperature some disintegrants are known to form a gel when they become hydrated (*i.e.* in CCS an increase in temperature promotes hydrogel formation) [94–96]. Any gel phase is formed *in situ* and will directly fill the macropores of the disintegrating matrix and thus slows down the liquid penetration.

**Fig. 7:**
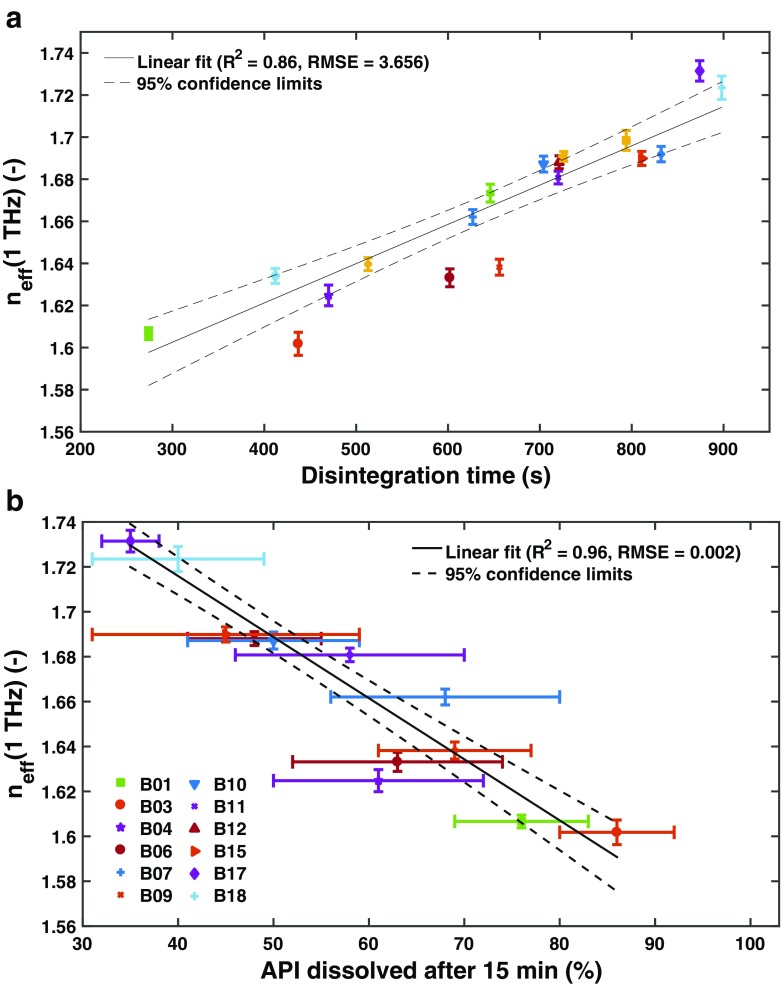
The effective refractive index, *n*_eff_, measured by THz-TDS as a function of the (**a**) disintegration time and (**b**) the amount of API dissolved after 15 min. The measurement was performed in transmission as schematically illustrated in Fig. 5. Samples of 18 batches of biconvex tablets from a production-scale design of experiments study into exploring the design space of a commercial tablet manufacturing process were used (modified from [86]).

Whilst the performance of a tablet is strongly influenced by the raw material properties [97] it is important to highlight the significant impact of the processing route and the processing parameters on the dosage from microstructure, and in turn the disintegration behaviour [14, 98]. Markl *et al.* [86] recently presented results of a study of immediate-release tablets where the impact of changes in process parameters on the disintegration and dissolution performance was analysed in detail (Fig. 7). The tablets were produced by a high-shear wet granulation process, fluid-bed drying and subsequent compaction. The pore structure of the tablets was analysed by THz-TDS. Since the formulation was the same for all batches, the variations in disintegration (disintegration time ranges from 280 s to 900 s) and dissolution (API dissolved after 15 min ranges from 35% to 85%) performance originated solely from the microstructure of the tablets. In this study the disintegrant was incorporated in the matrix *intra*- and *inter*-granularly. It is well know that the mode of consolidation of the excipients and the API, namely *intra*-, *inter*-granularly or in both phases, can impact the disintegration behaviour of a tablet [61, 99–105]. Therefore, the effectiveness of an excipient and especially of a disintegrant in different modes of incorporation can be significantly affected by the granulation technique and its configuration.

## QUANTIFYING DISINTEGRATION MECHANISMS

Most quantitative studies to date have either focused on measuring the swelling of single particles that are used as pharmaceutical excipients or on measuring the increase in volume of the entire dosage form during disintegration. For example Rudnic *et al.* [106] observed wetting and swelling of individual disintegrant particles using a microscope. They found that the rate and extent of swelling for any given type of disintegrant varied with particle size, *i.e.* larger particles showed substantially greater rates and extent of swelling compared to smaller particles. However, the contribution of the disintegrant particle size to total disintegrant action was found to depend on the particle size distribution (polydisperse vs monodisperse) of all excipient(s) and API(s) [107]. In a polydisperse formulation, small particles can fit within the pores between large ones and thus hinder the liquid from penetrating the powder compact and resulting in increased disintegration time. Formulations based on polydisperse particles furthermore increase the interparticulate bonding surface area (Fig. 2) which results in an increased tensile strength and thus may prolong the disintegration of such powder compacts. Clear understanding of tablet disintegration mechanisms can only be developed by investigating the entire powder compact and considering its formulation alongside its microstructural properties.

**Fig. 8:**
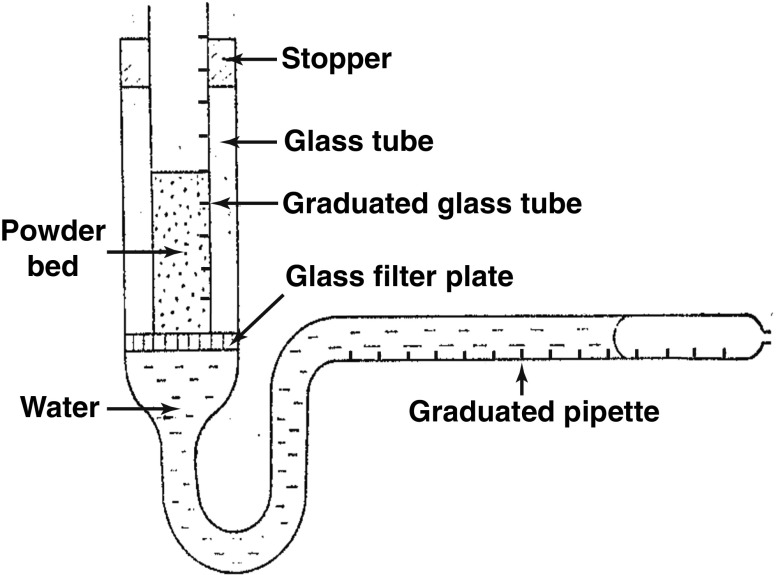
Apparatus to measure water uptake and swelling of a powder bed (modified from [108]).

Several studies were performed to measure water uptake into powder beds based on the apparatus presented by Nogami *et al.* [108] (Fig. 8). The water uptake of the powder bed was measured volumetrically by a graduated pipette and the swelling was recorded by reading the changes on the graduated glass tube. The same group also investigated the penetration rate of water into a powder bed. A certain amount of powder was packed in a graduated tube, which was then immersed in a thermally controlled beaker. The penetration front of the water into the packed powder was recorded and analysed on the basis of the Washburn equation.

Gissinger and Stamm [109] used the device shown in Fig. 8 to investigate the dependence of the water uptake on the wettability of a broad range of disintegrants. They emphasised that disintegration is accelerated for materials that exhibit a small contact angle, which is also in agreement with Eq. 1 indicating that a smaller contact angle leads to a larger volumetric flux. They further measured the swelling of tablets of pure disintegrants during the water uptake measurement using a linear inductive transducer. The authors concluded that an investigation of the disintegration action has to consider wettability (contact angle), water absorption and swelling capability of the powder compact.

A systematical characterisation of various formulations including different disintegrants and also for different microstructural properties was conducted in the 1980s on the basis of analysing the disintegration force (in the literature also known as the swelling force) as a function of time. For example, Colombo *et al.* [110] studied the effect of model substance properties, the properties and quantity of disintegrant, viscosity and temperature of the solvent and compression force on the disintegration force-time measurements. The authors indicated that the higher the model substance hydrophilicity, the lower the expansion rate constant and thus it was concluded that the diffusion process slows down the tablet expansion process. Moreover, it was found that the expansion rate constant decreases with increasing viscosity of the solvent and with increasing compression force (*i.e.*, reduction of the tablet porosity) and thus both cases prolong the disintegration time. Various other methods [40, 111–114] have been developed to study the mechanical force-time curves during disintegration by recording the swelling force exerted by the tablet against a fixed barrier. These measurements were then related to the structure of the tablet. Some of the studies [115, 116] analysed the data on the basis of a Weibull distribution, which was introduced to the pharmaceutical community by Langenbucher [117] to linearise dissolution curves. The Weibull distribution was found empirically to analyse most common dissolution data by a few characteristic parameters. The distribution can be expressed as

3 $$ \log\ \left[- \ln\ \left(1-\frac{F}{F_{\infty }}\right)\right]= b \log\ \left( t-{t}_0\right)- \log\  a. $$

*F* is the disintegration force at time *t* and *F*_∞_ is the maximum disintegration force. The parameters *a*, *b* and *t*_0_ were estimated from the best fit of the experimental data. Furthermore, replacing the parameter *a* = (*τ*_*d*_)^*b*^ provides the more informative parameter *τ*_*d*_, which represents the time needed to obtain 63.2% of the maximum disintegration force (measured from the end of the lag time *t*_0_). Colombo *et al.* [115] introduced an input value, *i.e.* the disintegration force development rate at time *t* = *t*_0_ + *τ*_*d*_, which is very sensitive to the formulation and structural changes of the tablet. They further revealed a good correlation between the input value and disintegration time.

**Fig. 9:**
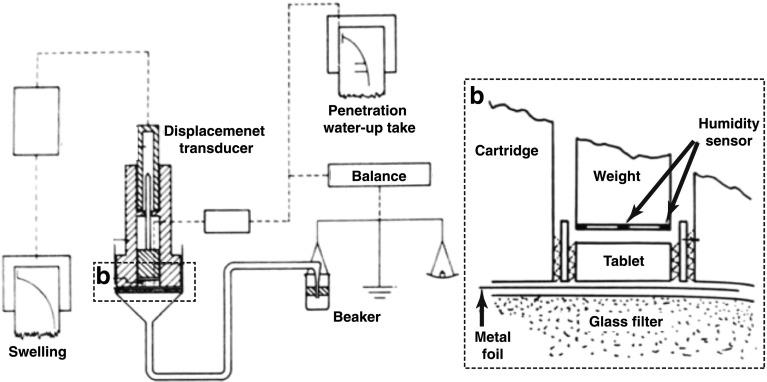
An apparatus to determine water penetration, water up-take and swelling of a tablet simultaneously. The tablet is placed upon a thin metal foil on a glass filter. The upper face of the glass filter is on the same height as the water level in the beaker (modified from [118])

Since liquid penetration, swelling and dissolution influence each other, it is necessary to measure and quantify each aspect individually in order to gain insights into their complex interplay. Dees [118] developed an apparatus to determine water penetration, water up-take and swelling simultaneously (Fig. 9). The measurement was started by removing the metal foil between the glass filter and the dry tablet sample resulting in the wetting of the tablet. The amount of water absorbed by the tablet can be measured by the microbalance. The swelling of the tablet is recorded by the inductive displacement transducer. The apparatus is also equipped with humidity sensors to detect the time when the water reaches the upper tablet face. The penetration depth was calculated from the swelling by assuming that the water moves throughout the tablet as a horizontal front and that the effectiveness of swelling is constant across the entire tablet.

**Fig. 10:**
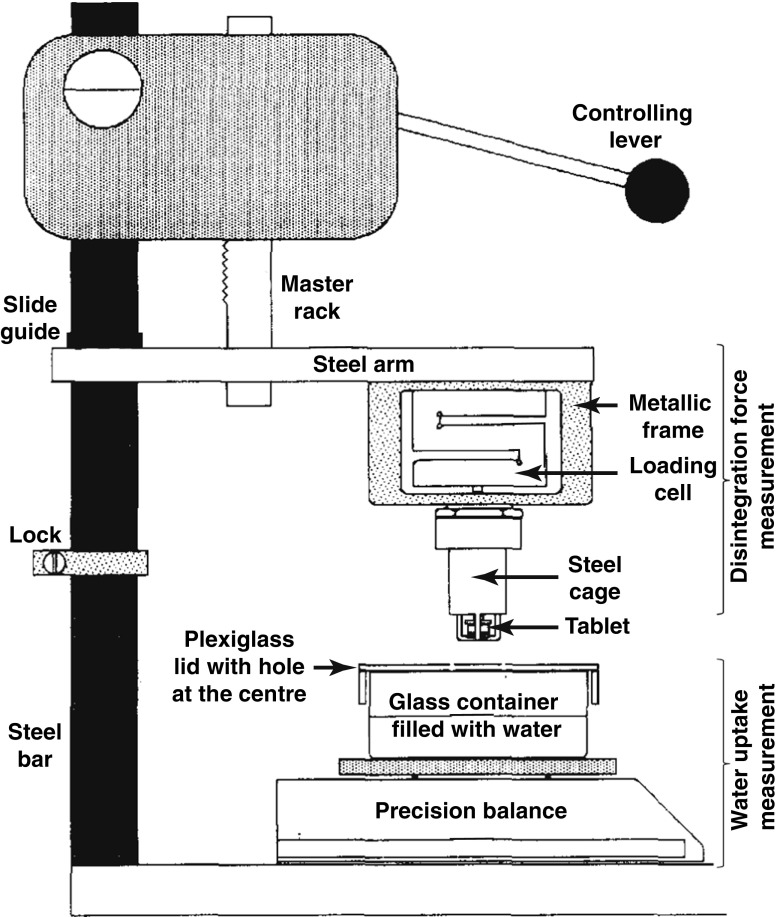
Apparatus to measure the disintegration force and the water uptake of a tablet. The tablet is clamped between the punch tip and the glass disk (modified from [112]).

Catellani *et al.* [112] measured simultaneously the amount of water absorbed and the force developed by the same tablet during its disintegration (Fig. 10). The principle for determining the amount of absorbed water is based on measuring the mass of fluid displaced by the tablet which corresponds to the upward thrust caused by a body immersed in a fluid. The tablet is pressed against the glass disk of the cage where the punch linked to the extensimetric loading cell which allows the measurement of the swelling force. The same device design was used to study the effect of pH and ionic content [119, 120] and to analyse the shapes of the disintegrating force *versus* time curves. The shapes of the force *versus* time ranged from a skewed distribution curve to a bell-shaped curve, depending on whether slow or rapid disintegration of tablets dominated, respectively. In order to compare different disintegrants, Caramella *et al.* [121] combined the disintegration force and water uptake measurements to one single parameter, *i.e.* force-equivalent parameter. This parameter expresses the maximum capability of a swelling agent to transform water into a force and it was used to characterise the efficiency of disintegrant swelling. Bell and Peppas [122] developed another apparatus to investigate the swelling behaviour of crosslinked hydrophilic polymers under an applied load as a function of time and absorbed weight. The results indicated that the swelling capacity is a function of the polymers’ degree of crosslinking.

Using the swelling force and water uptake measurements, it was possible to relate different disintegrants to specific disintegration mechanisms, *i.e.* swelling mechanism for SSG and CCS, and the strain recovery mechanism for XPVP [113]. These findings were supported by a study from Desai *et al.* [43], who applied high-speed video imaging to visualise the disintegration and wetting of free disintegrant particles and compacts. They concluded that there was no significant swelling associated with XPVP in free and compacted particles. However, the effect of compression force on the disintegration of compacts containing XPVP strongly indicated that strain recovery is the major mechanism for XPVP disintegrant action. It was further shown on the basis of force and water uptake measurements that disintegration times of tablets with a swelling disintegrant are only slightly affected by relative tablet density, whereas the strain recovery disintegrant requires high relative densities for rapid disintegration [123]. The water uptake rate is in particular influenced by the permeability of the powder compact as discussed in the previous section.

**Fig. 11:**
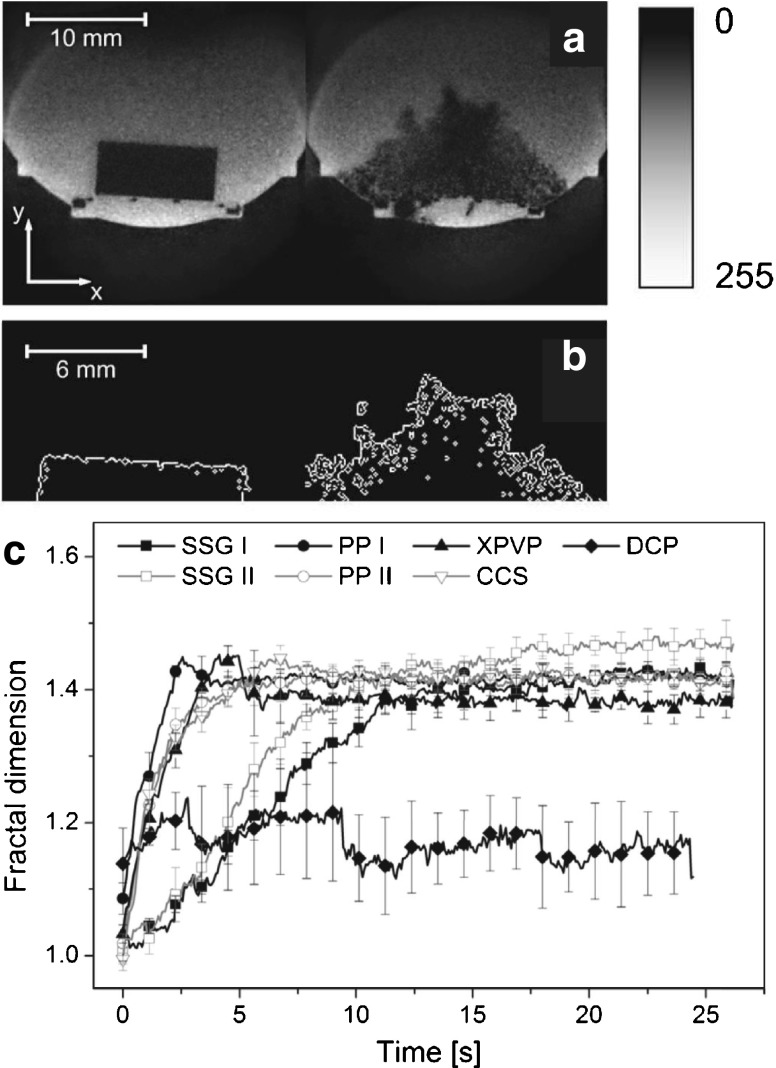
Quantitative analysis of tablet disintegration by MRI. (**a**) shows the MRI data, which is binarised using a threshold image intensity. Subsequently the edges are detected as depicted in (**b**), which is used for the calculation of the fractal dimension. This procedure was applied on MRI measurements of tablets with different disintegrants and analysed as a function of time as depicted in (**c**). SSG I: sodium starch glycolate (Explotab); SSG II: sodium starch glycolate (Primojel); PP I: polacrilin potassiumPolacrilin potassium (Amberlite IRP88); PP II: polacrilin potassium (Kyron T-314); XPVP: crospovidone (Polyplasdone XL), CCS: croscarmellose sodium (Ac-Di-Sol); DCP: dibasic calcium phosphateDibasic calcium phosphate (Di-Cafos C92-14) (modified from [131]).

In order to simultaneously study the penetration of liquid, microstructural changes and swelling, one needs to adequately visualise the process of disintegration from within a tablet in a non-destructive and contactless manner. Magnetic resonance imaging (MRI) was used very successfully to generate cross-sectional images of modified-release tablets during the exposure to liquid [124–127] and thus it was primarily used to study slow mass transport and swelling kinetics over a time scale of hours. However, Tritt-Gloc and Kowalczuk [128] employed dynamic MRI to study the disintegration behaviour of paracetamol tablets *in-vitro* under acidic gastric pH conditions. They employed an MRI system with an in-plane resolution of 117 × 117 μm^2^ and a section thickness of 200 μm. The authors estimated disintegration profiles on the basis of the MRI images for different commercial tablets containing paracetamol and for different fluid temperatures. Recent advances in high-resolution real-time MRI [129, 130] enabled the recording of MRI videos of disintegrating tablets (Fig. 11a) with a temporal resolution of 75 ms, a spatial resolution of 80 × 80 μm^2^ and a section thickness of 600 μm as presented by Quodbach *et al.* [44]. The improvements in terms of acquisition speed and resolution enabled a more detailed analysis compared to the setup presented by Tritt-Gloc and Kowalczuk [128]. The quantitative evaluation of the MRI data was performed on the basis of the grey value distribution of each image yielding information about the distribution and relative amount of water within a tablet during disintegration. This analysis was applied to differentiate the disintegration action of different disintegrants, where the results indicated differences between SSG (swelling), CCS (swelling), polacrilin potassium (PP, swelling) and XPVP (strain recovery) disintegrants [44]. The same group also presented an alternative data processing method of the MRI data [131], which calculates fractal dimensions of tablet boundaries (Fig. 11b and c). The fractal dimension is directly related to the surface area of a tablet and thus provides information about the effectiveness of the disintegration. However, this method could not sufficiently differentiate between tablets of varying relative densities and it only covers the initial phase rather than the complete course of the disintegration process.

Relative density differences and elastic properties of tablets can be studied by means of non-destructive ultrasonic measurements [132]. Akseli *et al.* published several articles about the use of ultrasonic methods to analyse mechanical properties of tablets [133, 134] and recently they utilised ultrasonic measurements to predict the breaking force and disintegration time of tablets [135]. The authors applied machine learning concepts (neural networks, genetic algorithms, support vector machines and random forest) to predict the disintegration time from ultrasonic measurements and several other tablet properties (tablet diameter, thickness, weight, porosity and breaking force) as well as process parameters (compression force and tablet compaction speed). The use of such statistical models may provide high correlation results, but one has to be careful when training such models to avoid overfitting and to assess generalisability. Moreover, statistical models do not reflect physical properties of the powder compact and thus no fundamental insights about disintegration phenomena can be gained from such models. However, the use of the ultrasound technique provides some very interesting insights into the internal structure of tablets and can be used as a very powerful sensor for in-die measurements during compaction process development [136, 137].

**Fig. 12:**
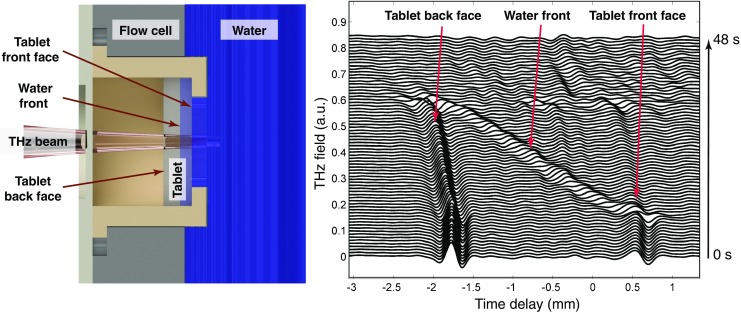
Schematic of the experimental setup for the *in-situ* monitoring of the disintegration process by TPI and the deconvolved time-domain terahertz waveforms (each line is offset by 0.01 a.u.). The terahertz waveforms shows the hydration of a pharmaceutical tablet consisting of 44% lactose anhydrous (Tablettose 100, Meggle, Wasserburg, Germany), 30% MCC (PH102, FMC Biopolymers, Philadelphia, USA), 20% paracetamol, 5% CCS, 1% magnesium stearate (MgSt) with 10% porosity.

A promising new technique to measure tablet disintegration is terahertz pulsed imaging (TPI). Most pharmaceutical excipients are transparent to terahertz radiation (far-infrared and sub-millimetre regime of the electromagnetic spectrum). In TPI short pulses of this radiation are focused on the dosage form of interest and the reflected echoes are recorded as a function of their time-of-flight, much like ultrasound or radar experiments [138]. Given the transparency of the tablet matrix to terahertz radiation information from both surface and internal structure of the dosage form can be measured in the same experiment. The terahertz pulse can propagate through the entire dosage form and reflections will be detected at every interface where the refractive index of the medium is changing such as internal cracks or the liquid front of penetrating liquid into the tablet [139, 140]. This principle enables the monitoring of the swelling and the liquid ingress as shown in Fig. 12 [77]. Yassin *et al.* [77] demonstrated that using this technique it is possible to analyse liquid ingress and tablet swelling quantitatively. In addition, it is possible to detect cracks that can form in some matrices due to the strain exerted by the hydration. Given the measurements are fast (acquisition rates of less than 10 ms have previously been reported [141]) the method is very well suited to investigate the disintegration of immediate-release tablets.

**Fig. 13:**
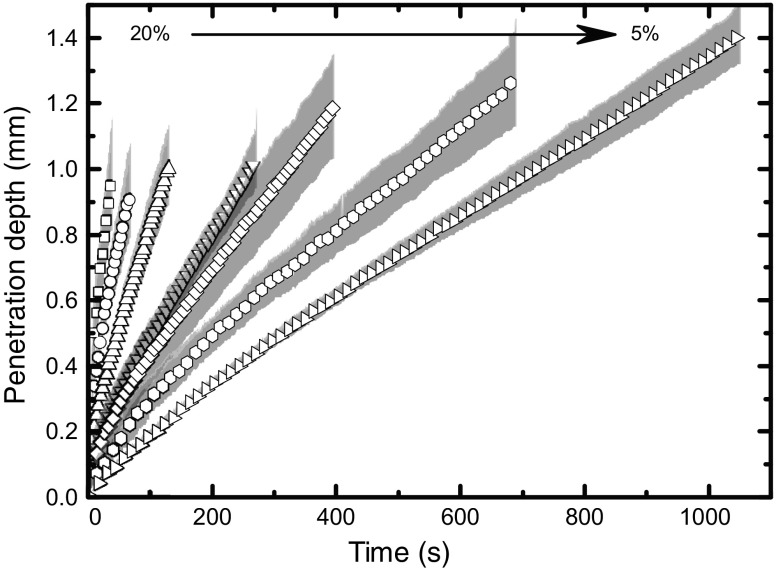
Disintegration kinetics quantified from TPI measurements of tablet samples containing MCC, lactose, MgSt and CCS with different porosities (ranging from 5% to 20% as indicated by the *arrow*). All samples were hydrated with water at a temperature of 20°C. The shading marks the standard deviation between individual experimental repeats.

Using the TPI method the effect of porosity and water temperature on the disintegration was investigated in detail. Liquid penetration and swelling was consistently faster at 37 °C compared to 20 °C [77]. The rates of swelling and wicking were found to correlate with the porosity of the tablet and could be described by a simple Darcy flow model (Fig. 13).

In a further study the effect of different binders, lubricants and disintegrants was studied [142]. Lubricants are highly hydrophobic materials, which significantly affect the wettability of the porous matrix. Therefore, a lubricant is expected to retard water penetration and thus the onset of disintegration and dissolution [143, 144]. The mass fraction of the lubricant is a critical factor as a minimum amount is required to cover the surface of the particles and thus to fully exploit the functionality of the lubricant [145, 146]. Yassin *et al.* concluded that in the samples containing a lubricant the hydration mechanism was dominated by anomalous mass transport (*m* ≈ 1 in Eq. 4 for all three tested disintegrants (CCS, XPVP and SSG). Given that anomalous transport processes result in inconsistent hydration and disintegration kinetics, and thus increases batch variability, this regime is not an ideal hydration mechanism for tablet disintegration. The study further revealed that there is a critical concentration of binder for a tablet formulation which will change the tablet properties and dominate both the hydration and disintegration kinetics. However, more work is required to understand the relation of lubricant and binder concentration to tablet disintegration kinetics in more detail.

**Fig. 14:**
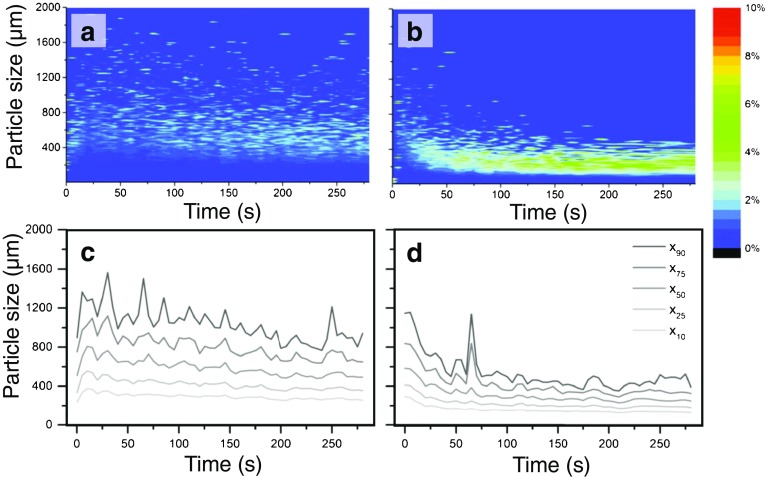
Particle size of disintegrated particles as a function of time measured by modified spatial filtering velocimetry. Left column is the data of SSG (Primojel, DMV-Fronterra Excipients, Goch, Germany) and the right one is the data of PP (AmberliteIRP88, Rohm and Haas France SAS, Chauny, France). (**a**) and (**b**) show particle size distributions (contour plots) of disintegrating tablets. The rows he colour codes are the relative percentage of a given particle size. The particle size developing over time is depicted in (**c**) and (**d**) for SSG and PP, respectively (modified from [154]).

The studies employing MRI and TPI primarily focused on the initial phase of tablet disintegration, *i.e.* liquid penetration, swelling and strain recovery, whereas the actual derupture of particle-particle bonds and the further detaching of particles from the tablet surface was not studied. Several research groups determined the particle size distribution of the detached particles directly. Shotton and Leonard [99, 100] used a combination of a wet sieving technique and a Coulter Counter to investigate the impact of *intra*- and *extra*-granular incorporation of the disintegrant. They concluded that *intra*-granular incorporation leads to smaller mean particle size, whereas the disintegration time was longer. In general, a smaller particle size causes a faster dissolution (see Eq. 13) which was investigated by employing focused beam reflectance measurements (FBRM) [147, 148] Focused beam reflectance measurements and dynamic laser diffraction [149–151]. Several other studies used video imaging to count the number of detached particles and to measure their size [20, 43, 152, 153]. Quodbach *et al.* [154] measured changes in particle size during the disintegration of tablets using modified spatial filtering velocimetry (Fig. 14). The detected particle size varied highly between the beginning and the end of the disintegration process and also between different disintegrants. They evinced that a further disintegration of particles occurs after break up of the tablet core, which was also indicated by Zhao *et al.* [151] using laser diffraction.

As highlighted in the previous section, the bioavailability of the dosage form can be significantly influenced by the GI environment. Several research groups investigated regional differences in the GI to gain more knowledge about the influence of theGI environment, as well as more predictable *in-vitro in-vivo* correlations [155]. The standard non-invasive methods are *γ* -scintigraphy labelled with *γ* -emitting radioisotopes [156, 157] or the visualisation of X-ray dense drug dosage forms by repetitive X-rays [158, 159]. However, the application of ionising radiation to humans is mostly restricted by national laws on radiation protection and therefore, there was the need to find alternative methods like biomagnetic techniques. Magnetic sensors used for such investigations typically employ induction coils to measure biomagnetic fields resulting from ferromagnetic sources in response to an applied magnetic field. Thus, the samples must be labelled by magnetic materials, which is achieved by the incorporation of powdered ferromagnetic substances (*e.g.* iron oxide) into the dosage form. The technique of magnetic marker monitoring combined with a very sensitive magnetic detector system, *i.e.* superconducting quantum interference devices (SQUIDs), has been used to record extremely sensitive magnetic fields with a high temporal and spatial resolution. The strength, the three dimensional localisation and orientation of the magnetic source can be reconstructed from these measurements as a function of time [160–162]. SQUIDs have been employed for the *in-vivo* characterisation of the oesophageal transit, the gastric and the intestinal behaviour of capsules [163, 164] and tablets [161]. These studies showed that the *in vivo* disintegration of capsules in the stomach correlates very well with the disintegration behaviour measured *in-vitro* [164]. However, they further observed in several cases in different volunteers that a capsule disintegrated in the small intestine and not in the stomach as intended. In these cases the gastric residence times were very short (< 3 min) and in one case a capsule remained only for 15 s in the stomach.

Similar studies were performed by applying multisensor alternate current biosusceptometry (ACB) to analyse the *in-vitro* and *in-vivo* disintegration performance of magnetic tablets in the human colon under normal physiological conditions [165]. These measurements enabled the quantification of the *in-vivo* performance by the gastric residence time, small intestinal transit time, orocaecal transit time and the disintegration time of magnetic formulations in the human gastrointestinal tract [165–167]. Cora *et al.* [168] further estimated disintegration properties as well as the kinetics of disintegration process for different compression forces combining ACB, water uptake and disintegration force measurements.

## DRUG RELEASE KINETICS

Traditionally the key parameter to assess the performance of a drug is to study the dissolution kinetics. As discussed above, dissolution might occur simultaneously with disintegration, though in the majority of cases one refers to the dissolution afterthe disintegration. However, disintegration and dissolution are interlinked and both processes have to be considered when one assesses and further wants to improve drug performance. Optimising the drug performance by modifying the disintegration processes is specifically important for the increasing number of poorly-soluble drug candidates, where dissolution is mainly the rate-limiting step in drug absorption [169, 170]. This section focuses on results from dissolution studies related to immediate-release tablets, which are readily impacted by disintegration.

**Fig. 15:**
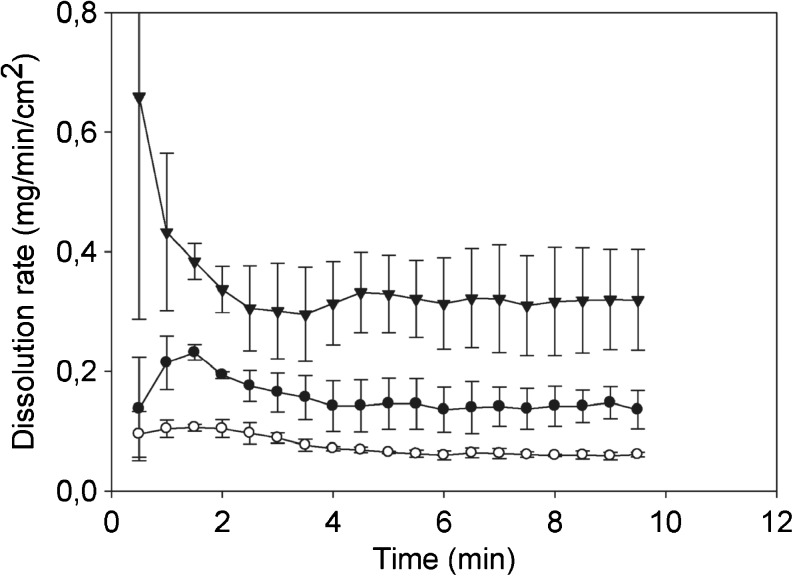
Dissolution rates of amorphous amlodipine besylate (▼), amlodipine besylate dihydrate (•) and amlodipine free base (∘) as a function of time using UV imaging (modified from [171]).

**Fig. 16:**
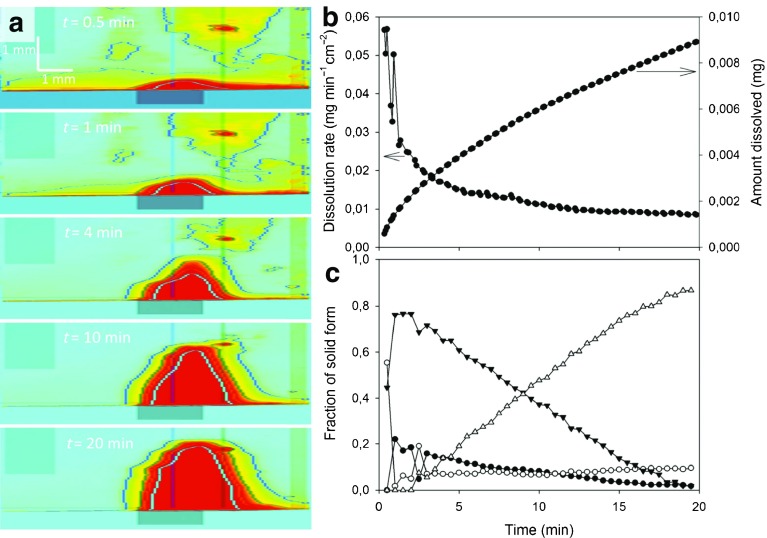
Studying the fast dissolution process of sodium naproxen in 0.1 M HCl by UV imaging and *in-situ* Raman spectropscopy. (**a**) UV absorbance maps for selected time points. Red colour indicates high absorbance and the contours represent isoabsorbance lines. (**b**) The dissolution rate and cumulative amount of dissolved naproxen. (**c**) Fractional sodium naproxen/naproxen form conversion as a function of time. The fractions of intermediate solid form 1, intermediate solid form 2, intermediate solid form 3, and naproxen are represented as (▼), (•), (∘) and (△), respectively (modified from [177]).

Traditional dissolution testing cannot be used to gain insights about the early dissolution events acting in parallel to the disintegration as these methods suffer from delayed response. The most promising technique to study early dissolution is ultra-violet (UV) imaging providing temporarily and spatially resolved absorbance maps. Bøtker *et al.* [171] combined UV imaging, Raman spectroscopy and a channel flow cell method to study the dissolution of amlodipine (Fig. 15). The authors demonstrated that the dissolution of amlodipine besylate was faster from the amorphous form than from the crystalline forms. It is well known in pharmaceutical sciences that the dissolution rate can be optimised by changing the solid-state properties of the drug. This includes the use of high-energy solid forms (*e.g.*, amorphous systems), solid dispersions or different polymorphs [172]. These approaches often result in metastable or even unstable forms of the API, which might convert to a thermodynamically more stable form [173] during disintegration. Therefore, it is of great importance to better understand the affect of a dissolution medium on the solid-state properties of the drug [174–176]. Østergaard *et al.* [177] applied simultaneously UV imaging with *in-situ* Raman spectroscopy for the characterisation of drug dissolution and solid state transformation during dissolution. They demonstrated that sodium naproxen (Fig. 16) converted to the more stable forms, *i.e.* naproxen monohydrate, within 5 min.

To date research focused on the *in-situ* analysis of modified-release tablets, where non-destructive methods including near-infrared (NIR) Near-infrared spectroscopy [7, 178], Raman spectroscopy [171, 177, 179], UV imaging [171, 177, 180, 181], infrared imaging [178, 182–184] and MRI [124, 185, 186] have been used very successfully. However, there is still a lack of understanding immediate-release tablets and solid state transformations occurring when the dissolution medium comes in contact with liquid.

## MODELLING OF DISINTEGRATION AND DISSOLUTION

The last sections highlight that significant progress was made experimentally in recent years to measure and better understand disintegration phenomena. In order to transform the design of solid dosage forms from an empirical art to a rational science it is essential to quantitatively describe the relationship between structure, formulation and disintegration behaviour. Mathematical models that accurately describe the physics of the process are required to reliably predict tablet disintegration, dissolution and eventually the drug release profile. In order to achieve this the models not only have to describe liquid ingress, swelling, strain recovery, dissolution as well as disruption of particle-particle bonds (Fig. 3) with sufficient accuracy but also how these processes are linked and interdependent. This is clearly a highly complex problem. Although several studies presented models for each mechanism independently, to the authors’ best knowledge, there is no single model combining the different phenomena.

### Liquid Penetration and Swelling

**Fig. 17:**
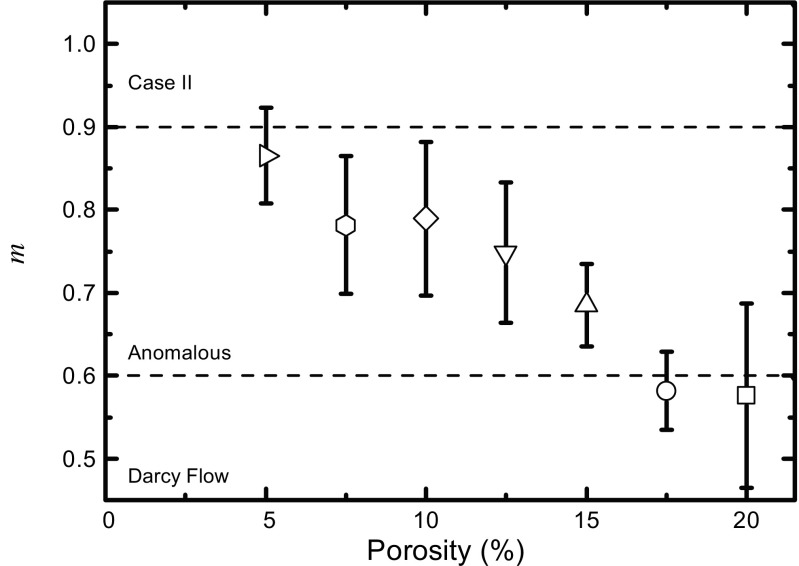
Exponent *m* (see Eq. **4**) estimated from TPI measurements depending on porosity. The liquid penetration of the respective samples are shown in Fig. 13. Samples contain MCC, CCS, lactose and MgSt. Tablets were hydrated at 20°C. *Error bars* represent the standard deviation.

The transport kinetics of a range of formulations and physical properties were modelled by Yassin *et al.* [77] using a simple power law. This semi-empirical model was previously used to study drug release kinetics [187–190] and can be expressed by

4 $$ y= k{t}^m, $$

where *k* is a constant related to the structural and geometric characteristics of the tablet and the exponent *m* is related to the mass transport mechanism [187]. In the study by Yassin *et al.* the parameters *k* and *m* were fitted to experimentally acquired time-resolved liquid penetration data as measured by TPI (see Fig. 13 for the data). Based on the value of the exponent *m* the mass transport mechanism is assumed to be dominated by a pressure gradient (typically referred to as Darcy flow), by an activity gradient (case II relaxation) or can be regarded as a combination of both (anomalous diffusion) [77]. Figure 17 shows the results of such analysis for a range of porosities in MCC based tablets. As expected, Darcy flow characteristics are dominating at higher porosity as faster liquid penetration can take place given the larger amount of available pore space.

The experimental results highlight that under certain conditions the movement of the liquid front in a porous tablet with porosity *ε* can be described by Darcy’s law (Eq. 18) [87]. An equation for the liquid penetration can be analytically derived by neglecting the effect of gravity and using the Young-Laplace equation (Eq. 16) for the capillary pressure:

5 $$ L=\sqrt{\varepsilon K\frac{4\gamma cos\ \theta}{\eta {R}_{c,0}} t}. $$

As a note, the capillary radius *R*_*c*,0_ is the pore radius, which is seen by the liquid meniscus. If the liquid penetration is driven by capillary action, it can also be modelled using the Hagen-Poiseuille (Eq. 15) and Young-Laplace (Eq. 16) equations. Combining both equation gives an expression for the liquid front *L* in the porous medium, which is known as the Washburn equation [191]:

6 $$ L=\sqrt{R_e\frac{\gamma cos\ \theta}{2\eta} t}. $$

Here *R*_*e*_ = *R*_*h*,0_^2^/*R*_*c*,0_ (*R*_*h*,0_ is the hydrodynamic radius) is the mean effective pore radius. The Washburn euqation is commonly used across a range of scientific and engineering disciplines to study penetration kinetics in porous media. One of the first applications of the Washburn equation in the pharmaceutical science was presented by Nogami, Hasegawa and Miyamoto [36]. They slightly adapted Eq. 6 to predict the water penetration time in aspirin tablets with starch as a disintegrant, which showed a good correlation with the measured disintegration time.

Both the Washburn equation and Darcy’s law approach conclude a square root dependence of the liquid penetration on time. Comparing the power law (Eq. 4) with Darcy’s law (Eq. 5) and the Washburn equation (Eq. 6) shows that both models predict the liquid penetration in powder compacts to follow the behaviour of *m* = 0.5 in Eq. 4. It further shows that the constant *k* in this expression is related to the microstructure of the matrix (porosity, pore radius, permeability), fluid (viscosity, surface tension) and fluid/matrix (contact angle) properties.

However, these simple models were developed for rigid systems and do not account for any swelling of the matrix during hydration. As discussed in the previous sections, swelling is not only very common for pharmaceutical formulations but it is often essential for successful disintegration to take place. Swelling results in a dynamic change of the intrinsic permeability, porosity and pore radius. It is therefore very useful in the pharmaceutical context to consider models that have been developed specifically to describe the hydration/dehydration of porous food materials [192] and wicking in paper-like porous media [193–195].

Schuchardt and Berg [196] adapted the Washburn equation by assuming a linear decrease with time of the pore radius in the wetted area of a porous medium (a composite of cellulose and superabsorbent fibres). They considered *R*_*h*_ as the time-dependent effective hydrodynamic radius behind the advancing liquid front. *R*_*h*_ is thus defined as a linear function of time (*R*_*h*_ = *R*_*c*,0_ − *a* ⋅ *t*), where *a* is the rate of pore radius constriction. The modified Washburn equation is thus expressed by

7 $$ L={\left(\frac{R_{c,0} cos\ \theta}{2\eta}\right)}^{1/2}{\left( t-\frac{a}{R_{c,0}}{t}^2+\frac{a^2}{3{R}_{c,0}^2}{t}^3\right)}^{1/2}. $$

Following the approach by Schuchard and Berg, Masoodi *et al.* [194] developed a model based on Darcy’s law in order to consider the swelling of the particles within the solid matrix and thus the constriction of the pores. They derived the following equation for the liquid penetration:

8 $$ L=\sqrt{\frac{2{P}_c}{\varepsilon_0\eta}{\displaystyle \underset{0}{\overset{t}{\int }}} K\left({t}^{\prime}\right) d{t}^{\prime }}. $$

This model allows to analyse the time-dependent permeability, porosity and pore size and thus it provides an insight into the microstructural changes during the hydration of swelling porous media. However, it is important to point out that these models describe the swelling process only during the transient liquid penetration and do not provide any details about the subsequent swelling once the powder compact is fully hydrated.

Experimental data of samples that contain a large amount of crosslinked polymer or microcrystalline polymer indicates that typically two phases of swelling are taking place successively in such materials: initial rapid swelling due to liquid penetration and secondary swelling due to the disentanglement and diffusion of the polymer macromolecules into the hydrating solution [45, 46]. The second, much slower, phase of swelling appears to be asymptotic in nature and can be modelled using the Schott model [45, 46]. The original Schott model was developed to describe the water uptake in semicrystalline polymers such as gelatine and cellulose expressed as a mass uptake in grams of absorbed solution per grams of solid matrix. It was modified for the case of one-dimensional swelling Δ*δ* (in units of m):

9 $$ \Delta \delta =\frac{t}{A+\frac{1}{\delta_{\infty }-{\delta}_0} t}. $$

*A* (swelling) is a material constant that can be determined experimentally by linear regression. *δ*_0_ and *δ*_∞_ represents the initial tablet thickness and the final thickness of the tablet after swelling, respectively. It was shown that 1/*A* is related to the initial swelling rate of the tablet, which could be further used to model the swelling of single particles and its impact on the pore radius reduction during the initial fast hydration process.

**Fig. 18:**
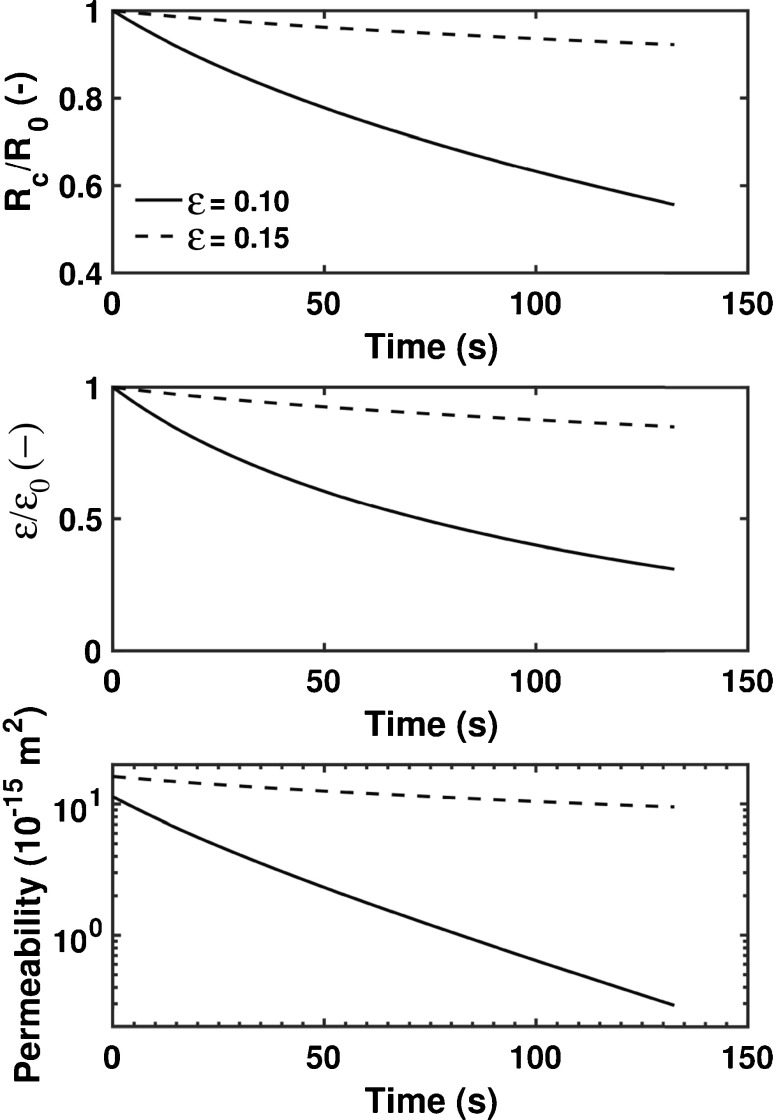
Simulation results from modelling the liquid penetration in swelling MCC tablets for two different tablet properties (solid line corresponds to *ε*_0_ = 0.10 and dashed line corresponds to *ε*_0_ = 0.15). *R*_0_: initial pore radius; *R*_c_: time-dependent pore radius; *ε*_0_: initial porosity; *ε* time-dependent porosity. A modified Carman-Kozeny (see Eq. (5) in [208]) was used to model the permeability as function of porosity.

The combination of the Schott model (Eq. 9) and the method based on Darcy’s law (Eq. 8) facilitates the analysis of microstructural changes during the liquid penetration, such as pore radius, porosity and permeability as a function of time (Fig. 18). As the data in the figure shows, significant changes of the porosity, pore radius and permeability can be observed for plain MCC tablets with two different initial porosities (*ε*_0_ = 0.10 and *ε*_0_ = 0.15). The swelling of individual MCC particles causes a decrease of the average pore radius, which reduces the porosity of the powder compact as time increases. Since the permeability is also a function of the pore radius, it decreases over time as well. These simulations clearly emphasise the complex interplay between the different microstructural properties of a tablet, which cannot be examined in such detail on the basis of experimental data only. However, newly developed models have to be validated by experimental data on the basis of characteristic measurable disintegration phenomena, *i.e.*, liquid penetration and swelling. Therefore, it is only through a combination of modelling approaches and *in-situ* monitoring that fundamental insights of the disintegration process can be gained.

**Fig. 19:**
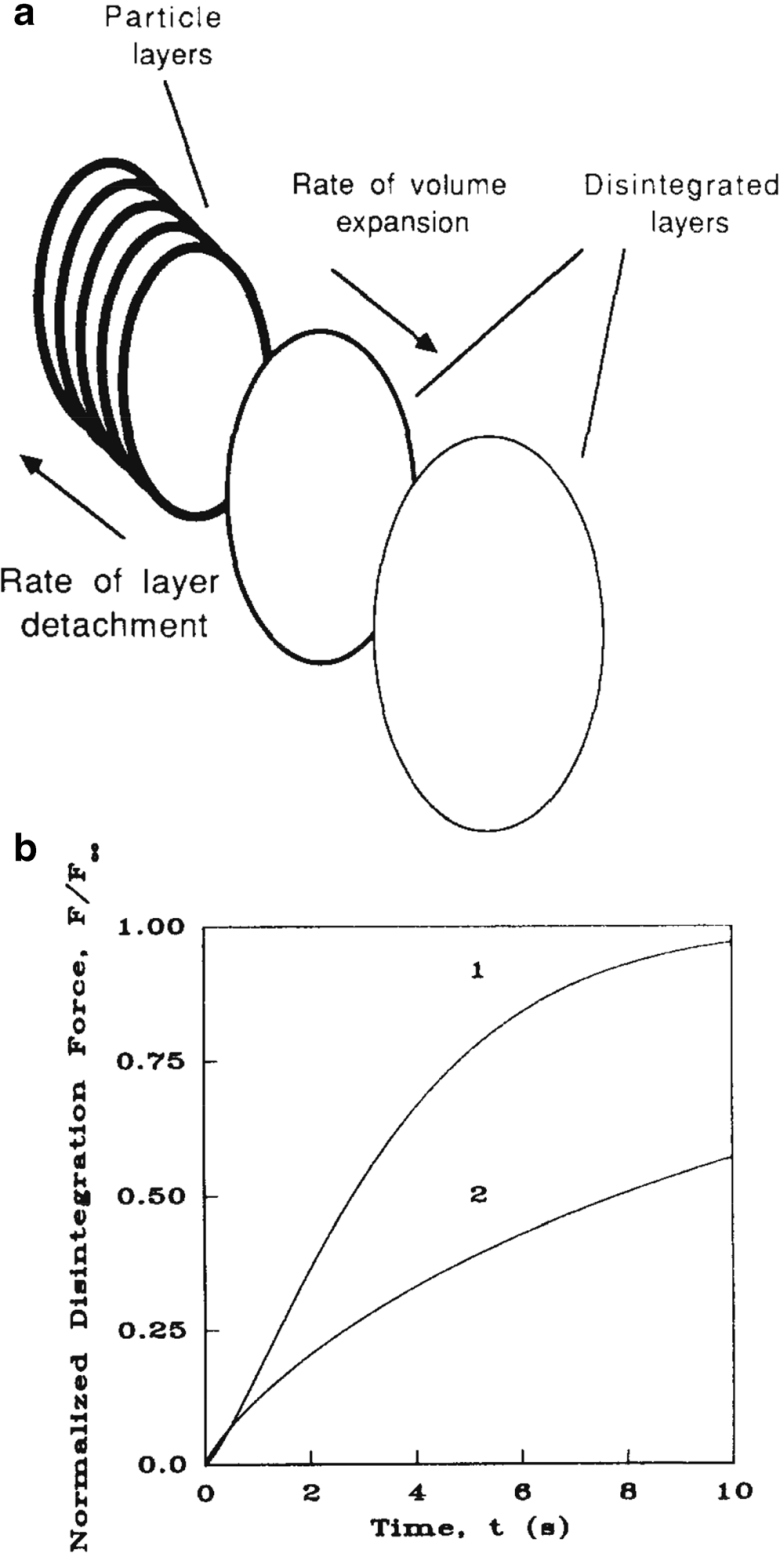
Modelling the detachment of particles during disintegraton. (**a**) Layer detachment and progressive tablet expansion. (**b**) Normalised disintegration (*swelling*) force depending on time. Curve 1 indicated the results for a tablet consisting of **CaHP**0_4_ ⋅ 2**H**_2_ **O** with 1 wt% polyplasdone XL. Curve 2 corresponds to **CaHP**0_4_ ⋅ 2**H**_2_ **O** with 20 wt% polyplasdone XL (modified from [197]).

Using a different approach, swelling and the resultant detachment of particles was modelled by Caramella *et al.* [197] by considering the disintegration mechanism as physically analogous to the phenomenon of nucleation and growth [40, 198]. Their model treats the progressive tablet expansion alongside an associated layer detachment process (Fig. 19). A similar model was already introduced in the 60s by Nogami, Hasegawa and Miyamoto [36] to study the liquid penetration into aspirin tablets. In the models of both groups the assumption is made that the disintegration of particles occurs only in layers parallel to the surface of the largest area of the tablet (*i.e.* in axial direction). In the model from Caramella *et al.* the normalised disintegration force as a function of time is given by the equation

10 $$ \frac{F}{F_{\infty }}=1- \exp\ \left(- a{t}^m\right), $$

with *a* as the expansion rate and where *m* is representing the mechanism of the disintegration. Caramella *et al.* [197] extracted and compared these parameters to differentiate between interface-controlled (*i.e.* interfacial detachment of particles from the surface) and diffusion-controlled (*i.e.* local diffusion of particles once they have been detached) disintegration. Peppas and Colombo [40] later expanded this analysis and provided a model which considers fluid mechanical phenomena, the changes in pore structure during the initial water uptake as well as the swelling of the disintegrant:

11 $$ F={C}_0+{C}_d{w}^{1/2}+{C}_c w $$

*w* is the amount of water entering the tablet expressed as grams of water per grams of solid matrix. *C*_0_ describes the initial stresses of the tablet and the potential change of stresses when water fills the pores. *C*_*c*_ and *C*_*d*_ are indicative for the relative importance of the convective and diffusive portion of the disintegration phenomenon. The model was verified using the apparatus presented in Fig. 10 and it was shown that the force is a linear function of the absorbed amount of water until the swelling becomes active and causes a change of the kinetics, i.e. the force is then a function of the square root of the water quantity.

The models by Caramella *et al.* were a first step towards describing the interruption of particle-particle bonds. In order to model the rupture of the *inter*-particle bonds, one needs to consider the formation of cracks within the tablet [142]. Cracks may propagate in the direction of fluid movement through the tablet until the critical crack length is reached where the dosage form fractures. This process is conceptually similar to the more well understood mechanisms in other fields of wet granular matter (pendular, funicular, capillary and slurry states). Therefore, models developed in these fields [199–201] could be used in future to quantitatively describe the last phase of the disintegration process and to determine the critical stage when the liquid bridges rupture and the tablet completely disintegrates.

### Drug Release Kinetics

The history of dissolution research started in the 19th century when Noyes and Whitney conducted the first dissolution experiments [202]. The authors concluded that the rate at which a solid dosage form dissolves is proportional to the difference between the instantaneous concentration *c*_*t*_ at time *t* and the concentration of the saturated solution *c*_*S*_ . This statement can be expressed as

12 $$ \frac{d{ c}_t}{ d t}= k\left({c}_S-{c}_t\right). $$

Nernst [203] and Brunner [204] carried out further experimental studies and addressed the physical meaning of the constant *k* as defined by Noyes and Whitney. They applied the diffusion layer concept and Fick’s first law yielding the Nernst-Brunner equation:

13 $$ \frac{d{ c}_t}{ d t}=\frac{DS}{V\delta}\left({c}_S-{c}_t\right). $$

*D* is the diffusion coefficient, *S* the surface area available for diffusion/dissolution, *δ* is the thickness of the diffusion layer and *V* is the volume of the dissolution medium. From looking at the Nernst-Brunner equation, it is immediately obvious that the kinetics of drug dissolution is affected by intrinsic and extrinsic factors. The intrinsic properties of a drug substance that may influence the dissolution include crystallinity, polymorphism, hydration, particle size and particle solid surface. The total surface area of the sample exposed in the solvent is one of the main aspects that influences the dissolution rate. In fact the dissolution process can be accelerated by increasing surface area and decreasing the particle size. Furthermore, hydrodynamics and composition of the dissolution medium (*e.g.* pH, temperature, presence of solubilising agents) are extrinsic factors which affect the kinetics of dissolution. The Noyes-Whitney and Nernst-Brunner equations provided the basis for understanding drug release kinetics; even though they do not address all mechanisms involved in the drug release process. Wilson *et al.* [153] used a slightly modified form of the Nernst-Brunner equation and combined it with a population balance model to simulate the dissolution profile by considering the erosion of a tablet. The authors considered disintegration and dissolution in terms of reaction rates enabling the combination of both processes.

As summarised by Siepmann and Siepmann [189], besides the dissolution process itself the drug release of oral dosage forms includes the diffusion of water into the system, drug diffusion out of the device, polymer swelling, matrix former erosion, osmotic effects and various other phenomena. These processes occur in sequence but differ in terms of action time. Although drug diffusion is the predominant step in the majority of the cases, polymer swelling or polymer degradation/matrix erosion need to be considered to fully understand the drug release kinetics.

The dissolution behaviour of controlled-release dosage forms was studied in much more detail by developing mathematical models and applying a range of non-destructive methods. A number of studies described the drug release kinetics by combining experimental data and theoretical models [188, 205]. It was shown that the rate of diffusion into and out of a tablet can be described by a semi-empirical equation, i.e. the power law as depicted in Eq. 4. *k* is again a constant related to the structural and geometric characteristics of the tablet; *m* is the release exponent indicating the mechanism of drug release. Another semi-empirical model is the Peppas-Sahlin equation [206] given as

14 $$ \frac{M_t}{M_{\infty }}={k}_1{t}^m+{k}_2{t}^{2 m}, $$

with the constants *k*_1_, *k*_2_ and *m. M*_*t*_ and *M*_∞_ are the absolute cumulative amount of drug released at time *t* and infinite time, respectively. Similar to the discussion above for porous systems, both the power law and the Peppas-Sahlin equation are used to differentiate between, here, Fickian diffusion and case II relaxation; Fickian transport relies on a concentration gradient and case II transport on an activity gradient. In analogy to our discussion above the power law can also be used to describe an anomalous diffusion containing both Fickian and case II characteristics. Siepmann and Siepmann [189, 190] described models for a broad range of controlled-release devices including reservoir and matrix systems, which may or may not exhibit an initial excess of drug, and that are valid for a range of geometries: slabs, spheres and cylinders. However, the majority of studies have not considered the impact of the dosage form’s microstructure, which is particularly important for immediate-release formulations. Only the combination of models describing the liquid penetration, swelling, the formation of cracks and the break up of the tablet as well as the dissolution of the disintegrated particles will lead to a sound understanding of the disintegration and dissolution processes of immediate-release tablets.

## CONCLUSIONS

For more than 15 years there has been a concerted effort in the pharmaceutical community to improve the quality and consistency of pharmaceutical products by introducing a paradigm shift to how we innovate higher quality medicines. This has included the development of concepts such as QbD and process analytical technology (PAT) initiatives that aim to actively encourage in an in-depth understanding of processes and product characteristics that could be used to implement suitable control strategies to pharmaceutical processing. Significant progress has been achieved and advanced analytical methods are now routinely deployed to test chemical and physical quality attributes throughout drug product development and manufacturing.

However, not all areas of process understanding and quality testing have been equally transformed by this development. Even though there is clearly a longstanding interest in improving the rational understanding of the complex disintegration process that is well documented in the literature and innovative methodologies have been proposed to better measure the phenomena involved there has been no breakthrough yet in developing robust quantitative models of the process that could be used for the rational design of disintegrating dosage forms.

We believe that one of the factors that presently limits the development of a better understanding of the fundamental importance of disintegration can be found in the anachronistic disintegration test prescribed by the pharmacopoeia. Not only does the test fail to provide any insight into the physico-chemical changes that govern disintegration but, by defining the disintegration time as the time after which the last of six tablets fully disintegrates, the test result makes it hard, if not impossible, to resolve the subtle variations in microstructure that are critical for the process. The test was developed more than 80 years ago and the testing protocol has not changed very much over the years yet a large range of novel rapidly disintegrating formulations, dosage forms and new excipients have been developed in the interim and with this development the quality control requirements have changed. Whilst the disintegration test has served an excellent purpose since its inception it had the unfortunate side effect that too many pharmaceutical scientists now habitually assume that the disintegration test is a suitable test to investigate disintegration. It is important to highlight that this is not the case – it is a very good test to document compliance with a particular validation protocol required by the pharmacopoeia but it was never designed to help with the understanding of the complex process itself.

Besides the analytical testing procedure itself we have identified a range of scientific challenges that need to be addressed before mathematical models will be available that can be used as confidently to predict disintegration as it is possible for dissolution today. The role of the microstructure of the porous matrix on the disintegration mechanism and kinetics is clear and it is absolutely clear that subtle variations in processing parameters result in significant changes for the disintegration process. A detailed understanding of the interplay between process parameters, microstructure and disintegration behaviour will be critical for high quality immediate-release products manufactured by continuous processing with active feedback loops controlling the process.

Overall, the design of immediate-release dosage forms will greatly benefit from quantitative physical models of disintegration and we hope this review will stimulate fruitful discussion and encourage further work in this area to achieve this aim in the near future.

## Appendix A: Liquid Penetration in a Porous Tablet

The liquid penetration in highly porous immediate-release tablets is driven by capillary forces. Therefore, the pore space is approximated as a bundle of capillary tubes of varying diameter. Based on this assumption liquid ingress can be described by a Hagen-Poiseuille motion of liquid in the set of parallel capillary tubes. The volumetric flow rate *Q*_*i*_ in a tube *i* (*i* ∈ ℕ, *i* ≤ *N* with *N* as the total number of cylindrical tubes) with uniform radius *R*_*h*,*i*_ (also known as the hydrodynamic radius) can be expressed by

15 $$ \begin{array}{l}{Q}_i{\left.=-\frac{\pi {R}_{h, i}^4}{8\eta}\frac{dP}{dx}\right|}_{x={L}_i}\hfill \\ {}=-\frac{\pi {R}_{h, i}^4}{8\eta}\frac{\varDelta {P}_i}{L_i}=-\frac{{\displaystyle {\sum}_{j=1}^M}\pi {\omega}_j{R}_{h, j}^4/{L}_j\varDelta {P}_j}{8 A\eta}\hfill \end{array} $$

where *L*_*i*_ is the length of the liquid front in tube *i* at time *t* and *η* is the viscosity of the liquid. Δ*P* is the total effective pressure which is acting to force the liquid along the capillary and consists of atmospheric pressure, hydrostatic pressure and capillary pressure. We could divide the pores in *M* pore size classes, where *ω*_*j*_ represents the number of pores in the *j*th pore size class (*j* ∈ ℕ, *j* ≤ *M*) with radius *R*_*h*,*j*_ and volumetric flow rate *Q*_*j*_. The total volumetric flux *q* = *Q*/*A* (with *A* as the cross-section area) is then given by

$$ q=\frac{{\displaystyle {\sum}_{j=1}^M}{\omega}_j{Q}_j}{A}. $$

Using the Young-Laplace equation for the capillary pressure

16 $$ \Delta {P}_j=\frac{2\gamma cos\ \theta}{R_{c, j}}, $$

where *γ* is the surface tension, *θ* is the contact angle, *R*_*c*,*j*_ is the capillary radius and neglecting the atmospheric pressure and hydrostatic pressure, yields

$$ q=-\frac{\gamma cos\ \theta}{4\eta}{\displaystyle \sum_{j=1}^M}\frac{\pi {\omega}_j}{A}\frac{R_{h, j}^4}{R_{c, j}}\frac{1}{L_j} $$

We further introduce the tortuosity factor *τ* to account for the lengthened distance of pores. *τ* is the ratio of the actual path along the pore to the straight flow path. The volume fraction of pores (approximated as the area fraction) is considered by

$$ \Delta {\beta}_j\approx \frac{\omega_j\pi {R}_{h, j}^2}{A}. $$

The volumetric flux *q* of the fluid can now be written as

17 $$ q=-\frac{1}{4\tau}\frac{\gamma}{\eta} \cos\ \theta {\displaystyle \sum_{j=1}^M}\Delta {\beta}_j\frac{R_{h, j}^2}{R_{c, j}}\frac{1}{L_j}. $$

## Appendix B: Darcy’s law

Darcy’s law can be expressed as

18 $$ {\left. q=-\frac{K}{\eta}\frac{dP}{dx}\right|}_{x= L}=-\frac{K}{\eta}\frac{\varDelta P}{L}, $$

with *K* as the intrinsic permeability. Comparing Eqs. 17 and 18,

$$ \begin{array}{l} q=-\frac{K}{\eta}\frac{\varDelta P}{L}=-\frac{1}{8\tau}{\displaystyle \sum_{j=1}^M}\Delta {\beta}_j{R}_{h, j}^2\frac{\varDelta {P}_j}{L_j}\hfill \\ {}=-\frac{1}{8\tau}{\displaystyle \sum_{j=1}^M}\Delta {\beta}_j{R}_{h, j}^2\frac{\varDelta P}{L},\hfill \end{array} $$

and using $\Delta P=1/ M{\displaystyle \sum_{j=1}^M}\Delta {P}_j$ and $L=1/ M{\displaystyle \sum_{j=1}^M}{L}_j$ gives an expression for the intrinsic permeability:

19 $$ K=\frac{1}{8\tau}{\displaystyle \sum_{j=1}^M}\Delta {\beta}_j{R}_{h, j}^2. $$

## ABBREVIATIONS

*∆P*: Total effective pressure
*∆β*: Pore size distribution
*∆δ*: Swelling
*A*: Material constant (swelling)
*a*: Expansion rate
ACB: Alternate current biosusceptometry
API: Active pharmaceutical ingredients
*C*_0_: Initial stresses of the tablet
*C*_C_: Convective portion of the disintegration
CCS: Croscarmellose sodium
*C*_d_: Diffusive portion of the disintegration
*c*_S_: Concentration of the saturated solution
*c*_t_: Instantaneous concentration
*D*: Diffusion coefficient
DCP: Dibasic calcium phosphate
*F*: Disintegration force
*F*_∞_: Maximum disintegration force
FBRM: Focused beam reflectance measurements
GI: Gastrointestinal
*K*: Permeability
*k*: Constant
*L*: Penetration depth
*M*_∞_: Absolute cumulative amount of drug released at infinite time
MCC: Microcrystalline cellulose
MgSt: Magnesium stearate
MRI: Magnetic resonance imaging
M_t_: Absolute cumulative amount of drug released at time t
*n*_eff_: Effective refractive index
NIR: Near-infrared
PAT: Process Analytical Technology
PP: Polacrilin potassium
*q*: Volumetric flux
QbD: Quality by Design
*R*_c_: Capillary radius
*R*_e_: Mean effective pore radius
*R*_h_: Hydrodynamic radius
*S*: Surface area
SQUID: Superconducting quantum interference devices
SSG: Sodium starch glycolate
*T*_g_: Glass transition temperature
THz-TDS: Terahertz time-domain spectroscopy
TPI: Terahertz pulsed imaging
UV: Ultra-violet
*V*: Volume of the dissolution medium
*w*: Amount of water entering the tablet
*x*: API mass fraction
XPVP: Crospovidone
XμCT: X-ray computed microtomography
*γ*: Surface tension
*δ*: Thickness of the diffusion layer
δ_0_: Initial tablet thickness
δ_∞_: Thickness of the tablet after swelling
*ε*: Porosity
*η*: Viscosity
*θ*: Contact angle
*τ*: Tortuosity
